# Restriction of cytosolic sucrose hydrolysis profoundly alters development, metabolism, and gene expression in Arabidopsis roots

**DOI:** 10.1093/jxb/eraa581

**Published:** 2020-12-30

**Authors:** Cristina Pignocchi, Alexander Ivakov, Regina Feil, Martin Trick, Marilyn Pike, Trevor L Wang, John E Lunn, Alison M Smith

**Affiliations:** 1 John Innes Centre, Norwich Research Park, Norwich, UK; 2 Max Planck Institute of Molecular Plant Physiology, Wissenschaftspark Potsdam-Golm, Am Mühlenberg, Potsdam-Golm, Germany

**Keywords:** Arabidopsis, hexose, neutral invertase, root, root transcriptome, sucrose, sugar signalling

## Abstract

Plant roots depend on sucrose imported from leaves as the substrate for metabolism and growth. Sucrose and hexoses derived from it are also signalling molecules that modulate growth and development, but the importance for signalling of endogenous changes in sugar levels is poorly understood. We report that reduced activity of cytosolic invertase, which converts sucrose to hexoses, leads to pronounced metabolic, growth, and developmental defects in roots of Arabidopsis (*Arabidopsis thaliana*) seedlings. In addition to altered sugar and downstream metabolite levels, roots of *cinv1 cinv2* mutants have reduced elongation rates, cell and meristem size, abnormal meristematic cell division patterns, and altered expression of thousands of genes of diverse functions. Provision of exogenous glucose to mutant roots repairs relatively few of the defects. The extensive transcriptional differences between mutant and wild-type roots have hallmarks of both high sucrose and low hexose signalling. We conclude that the mutant phenotype reflects both low carbon availability for metabolism and growth and complex sugar signals derived from elevated sucrose and depressed hexose levels in the cytosol of mutant roots. Such reciprocal changes in endogenous sucrose and hexose levels potentially provide rich information about sugar status that translates into flexible adjustments of growth and development.

## Teaser

A reduction in cytosolic invertase in Arabidopsis roots changes the sucrose to hexose ratio, resulting in profound alterations of metabolism, growth, development, and patterns of gene expression.

## Introduction

Sucrose catabolism in the cytosol of non-photosynthetic cells generates hexoses, hexose phosphates, and UDP-glucose, the substrates for primary metabolism and hence cellular maintenance and growth. Three distinct groups of plant enzymes are capable of sucrose catabolism: sucrose synthases (SUSs), acid invertases, and neutral invertases. Acid invertases are confined to the cell wall and to vacuoles, so sucrose catabolism in the cytosol proceeds via isoforms of either SUS or cytosolic invertase (CINV). Four isoforms of SUS (SUS1–SUS4) and putatively five isoforms of neutral invertase are found in the cytosol of Arabidopsis cells (Barratt *et al.*, 2009; Wan *et al.*, 2018). We showed previously that elimination of all four of the cytosolic isoforms of SUS has little effect on plant growth under standard conditions, whereas elimination of two CINV isoforms strongly reduces growth rate and alters root anatomy (the *cinv1 cinv2* mutant; Barratt *et al.*, 2009; Barnes and Anderson, 2018). Reductions in CINV activity have similar effects in other, diverse species (e.g. *Lotus corniculatus*, Welham *et al.*, 2009; rice, Yao *et al.*, 2009). CINV thus appears to be the main enzyme responsible for sucrose catabolism in the cytosol.

The dramatic effect on Arabidopsis growth of loss of CINV1 and CINV2 could stem from restricted flux of carbon from sucrose into primary metabolism and energy generation—and hence starvation—and/or from modulation of the sugar signalling networks that coordinate carbon availability with growth processes including cell division and the biosynthesis of structural molecules. In general terms, high levels of sugars generate signals that promote growth, whereas sugar starvation promotes catabolism that maintains carbon availability and represses cell division and energy-consuming biosynthesis (e.g. Koch, 1996; Contento *et al.*, 2004; Price *et al.*, 2004; Nicolaï *et al.*, 2006; Osuna *et al.*, 2007; Usadel *et al.*, 2008; Cookson *et al.*, 2016).

A recent study of cell wall biosynthesis in the *cinv1 cinv2* mutant revealed reduced levels of cellulose and aberrations in the arrangement of cellulose microfibrils and underlying cytoskeletal elements. The authors suggested that deficient cellulose biosynthesis was at least partially responsible for stunted growth in *cinv1 cinv2* seedlings. Consistent with this idea, levels of UDP-glucose (the substrate for cellulose synthesis) were lower in *cinv1 cinv2* seedlings than in wild-type seedlings (Barnes and Anderson, 2018). The authors concluded that reduced carbon availability accounted for at least part of the cellulose deficiency phenotype, which in turn partly explained the slow and abnormal patterns of growth of the mutant. However other mutant phenotypes could not be explained by starvation. First, provision of exogenous sucrose or hexoses only partly complemented the cellulose biosynthesis and growth defects. Second, *cinv1 cinv2* seedlings contained levels of sugars and starch comparable with or higher than those of wild-type plants (Barnes and Anderson, 2018). These observations open up the possibility that growth defects in the *cinv1 cinv2* mutant may stem from abnormal patterns of sugar signalling, rather than directly from carbon starvation.

To shed further light on the causes of the root growth defects brought about by loss of CINV1 and CINV2, we made growth, transcriptome, and metabolite measurements on roots of young wild-type and *cinv1 cinv2* seedlings in the presence and absence of exogenous sugars. We show that, in addition to the defects identified by Barnes and Anderson (2018), *cinv1 cinv2* roots have reduced organ and cell size, altered patterns of cell division and differentiation in the meristem, and alterations in levels of primary metabolites and in the root transcriptome. Some of the defects are consistent with carbon starvation in mutant roots, but others cannot be explained from current understanding of either starvation or hexose/sucrose signalling. We suggest that the profound and widespread changes in the *cinv1 cinv2* mutant stem from altered cytosolic sucrose to hexose ratios, brought about by a reduced capacity for conversion of sucrose to hexose.

## Materials and methods

### Plants and growth conditions

Arabidopsis [*Arabidopsis thaliana* (L.) Heynh.] plants for crossing, genotyping, and shoot characterization were grown in compost at 20 °C and 70% humidity with 140–160 μmol quanta m^–2^ s^–1^ and 12 h light, 12 h dark. For root studies, seeds were surface-sterilized with chlorine gas and grown on vertical plates containing 1× Murashige and Skoog salt mixture and 0.8% (w/v) agar without sucrose at 20 °C with 60 µmol quanta m^–2^ s^–1^ and 16 h light, 8 h dark.

Insertion lines in the Columbia-0 background were: SALK_131881 (At1g72000, A/N-InvF), WiscDSLox466C11 (At1g22650, A/N-InvD), and SALK_097137 (At4g34860, A/N-Inv B). Homozygosity of the insertion lines was confirmed by PCR genotyping (primers are listed in Supplementary Table S1). Lines *PIN1p:PIN1-GFP*, *DR5p:GFP*, *PLT1p:PLT1-YFP*, *PLT2p:PLT2-YFP*, and *WOX5p:GFP* were crossed with *cinv1 cinv2* to generate reporter lines.

### Transient expression

Coding regions of *INV* genes were amplified with gene-specific primers (Supplementary Table S1) containing the Gateway attB1 and attB2 recombination sequences, cloned into pK7FWG2,0 [encoding a C-terminal enhanced green fluorescent protein (GFP) driven by the *Cauliflower mosaic virus* (CaMV) 35S promoter], transformed into *Agrobacterium tumefaciens* strain AGL1, and infiltrated into leaves of *Nicotiana benthamiana* (Goodin *et al.*, 2002). The presence of CINV–GFP fusion proteins in infiltrated regions of *N. benthamiana* leaves was checked 3 d after infiltration. Leaf extracts (50 mM Tris–HCl, pH 7.5, 150 mM NaCl, 5 mM EDTA, 2 mM DTT, 1.5% polyvinylpolypyridone) were clarified by centrifugation and the supernatant fractions subjected to SDS–12% PAGE. Blots of the gels were probed with a commercial antiserum to GFP (Torrey Pines Biolabs TP401, www.chemokine.com) and developed with a commercial anti-rabbit antiserum.

### Root measurements

Roots were digitally scanned and length measured with ImageJ software (http://rsb.info.nih.gov/ij/). Meristem size was determined as the number of cells in individual cell files contained within the meristematic zone, defined as the region of isodiametric cells between the quiescent centre (QC) and the cell that was twice the length of the immediately preceding cell.

### Microscopy

Roots were stained in 10 µg ml^–1^ propidium iodide (PI) for 2–5 min, rinsed, and mounted in water. Starch was visualized by staining with Lugol’s iodine solution for 10 min, rinsing, and mounting in chloral hydrate solution. Prior to differential interference contrast imaging, material was fixed in ethanol overnight at 4 °C then cleared in chloral hydrate solution. Confocal images were taken with a Leica SP5 II scanning microscope (Leica Microsystems; http://www.leica-microsystems.com/). GFP and PI were excited at 488 nm and emission was detected at 490–560 nm and 570–670 nm, respectively.

### RNA sequencing

Total RNA was extracted from roots of 4-day-old seedlings, using Tri-Reagent (Sigma; www.sigmaaldrich.com) following the manufacturer’s protocol. DNA was removed using the DNA-Free Kit (Life Technologies; www.lifetechnologies.com). RNA-Seq libraries were prepared using the TruSeq RNA sample preparation kit (Illumina; www.illumina.com/) following the manufacturer’s protocol. Samples were pooled together into two lanes of six samples each and sequenced on an Illumina HiSeq2000. Reads were quality trimmed using Cassava v1.8.2 (Illumina) with a minimum quality score of Q30, then aligned back to the TAIR 9 reference (TAIR9_cds_20090619) using Maq v0.7.1 (Li *et al*., 2008) at default settings. Read counts for each gene were quantified using an in-house Perl script (tag_counter.pl) on the alignment pileup files. A multidimensional scaling plot based on the 500 tags with the largest variation between the four treatments, performed with tools in RobiNA (Lohse *et al.*, 2012), provided evidence of the reliability of the data. Biological replicates were closely clustered, and each treatment was completely separated from the other treatments (Supplementary Fig. S1). Differential expression analysis was performed using RobiNA. The normalization and statistical evaluation of differential gene expression were performed using edgeR (Robinson *et al.*, 2010) with a false discovery rate (FDR) *q*-value cut-off of 0.05 and multiple testing correction (Benjamini and Hochberg, 1995).

### Functional classification and clustering analysis

Functional classification of genes employed the TAIR Gene Ontology (GO) annotations tool (https://www.arabidopsis.org/tools/bulk/go; Berardini *et al.*, 2004) and MapMan (https://mapman.gabipd.org/; Thimm *et al.*, 2004; Usadel *et al.*, 2009). GO term enrichment analysis was performed with the PANTHER Overrepresentation Test (release 20150430; http://amigo.geneontology.org/rte) using the Bonferroni multiple testing correction.

### Quantitative real-time PCR

Total RNA was extracted with a Plant RNeasy Mini Kit (Qiagen; www.qiagen.com), DNA was removed using the DNA-Free Kit (Life Technologies; www.lifetechnologies.com), and cDNA was synthesized with Superscript III Reverse Transcriptase (Invitrogen; www.invitrogen.com), following the manufacturers’ protocols. Oligonucleotides were designed with QuantPrime Software (www.quantprime.de) (Supplementary Table S1). PCR products were detected with the SYBR Green Jumpstart Mix (Sigma; www.sigmaaldrich.com) using a Chromo4 Real Time PCR System (Bio-Rad; www.bio-rad.com). The endogenous control was 18S rRNA (At2g01010).

### Metabolite assays

Roots or shoots were excised and frozen immediately in liquid nitrogen. Sugars and starch were extracted and measured enzymatically (Bläsing *et al.*, 2005); other metabolites were extracted and measured using high-performance anion exchange chromatography coupled to tandem MS (HPAEC-MS/MS: Lunn *et al.*, 2006, modified as in Figueroa *et al.*, 2016).

## Results

**Fig. 1.**
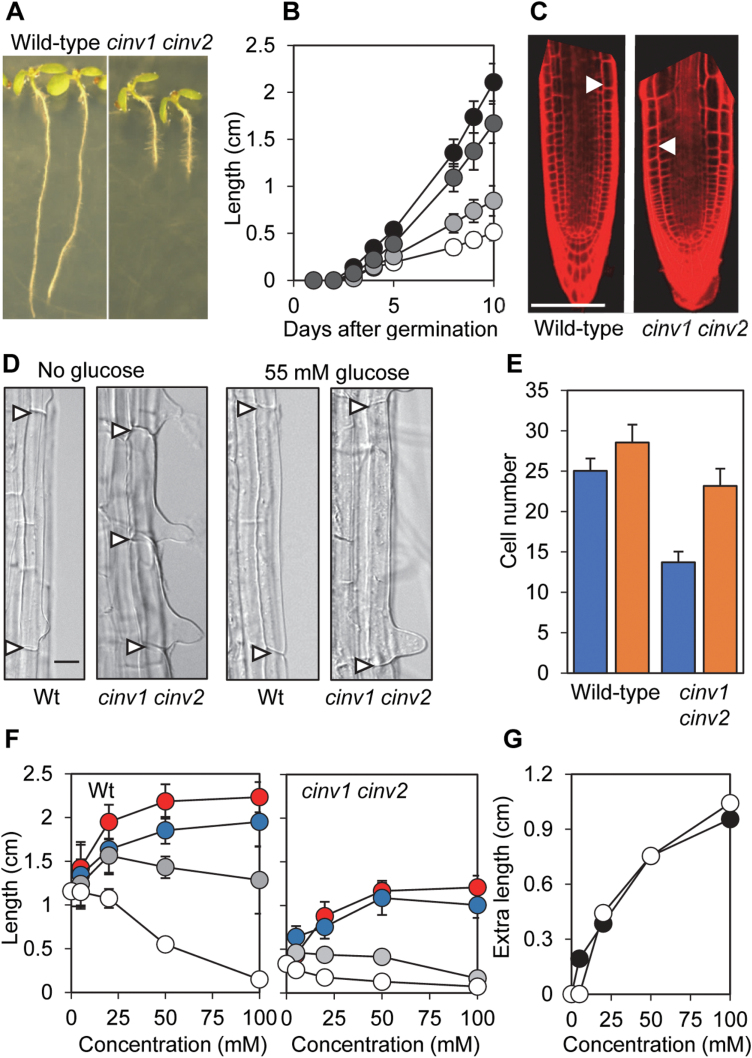
Reduced root length and apical meristem size in the *cinv1 cinv2* mutant. (A) Phenotype of 7-day-old seedlings grown vertically on medium without glucose. (B) Root length measurements of wild-type (black symbols), *cinv1* (light grey symbols), *cinv2* (dark grey symbols), and *cinv1 cinv2* (white symbols) seedlings on medium without glucose. Values are means of 25–30 measurements ±SD. (C) Confocal images of 4-day-old roots stained with propidium iodide. Wild-type (left) and *cinv1 cinv2* (right). Arrows indicate the boundary between the division zone (the meristem) and the elongation zone of the root (the point at which a given epidermal cell is twice the length of the cell immediately below). Scale bar=100 µm. (D) Lengths of mature epidermal cells in roots of seedlings grown with or without glucose. Arrows indicate walls between adjacent epidermal cells. Scale bar=10 µm. Cropped and reorientated images are shown for ease of comparison. The original, unedited micrographs used to make this composite are provided in Supplementary Fig. S2. (E) Meristem cell numbers in 4-day-old roots grown in medium without glucose (blue bars) or with 55 mM glucose (orange bars). Values are means of 50–60 measurements ±SD. For both genotypes, values with and without glucose were statistically significantly different, and for both treatments values for the two genotypes were statistically significantly different (Student’s *t*-test, *P*<0.0002). (F) Root lengths of wild-type (left graph) and *cinv1 cinv2* (right graph) seedlings grown for 7 d on different concentrations of glucose (red symbols), fructose (blue symbols), mannitol (grey symbols), and 3-*O*-methylglucose (white symbols). Values are means ±SD of 25–30 measurements. (G) Stimulation of root growth of 7-day-old seedlings by different concentrations of glucose. For each sugar concentration, the mean values for root length for 7-day-old wild-type (black symbols) and *cinv1 cinv2* (white symbols) seedlings grown on mannitol were subtracted from the equivalent value for roots grown on glucose [data from (F)] to give glucose-specific root extension.

Root growth in the *cinv1 cinv2* double mutant of Arabidopsis is severely reduced relative to that of wild-type plants (Fig. 1A; Barratt *et al.*, 2009; Barnes and Anderson, 2018). Most of the reduction in root length was due to loss of CINV1: loss of CINV1 alone had a relatively severe effect on root growth, and loss of CINV2 alone had only a minor effect (Fig. 1B; Lou *et al.*, 2007; Qi *et al.*, 2007; Barratt *et al.*, 2009). To check whether other CINV isoforms are individually important for normal root growth in Arabidopsis, we first confirmed the locations of three putatively cytosolic isoforms by transiently expressing them as C-terminal GFP fusions in leaves of *N. benthamiana*. The cytosolic location of isoforms encoded by At4g34860 (CINVB), At1g22650 (CINVD), At1g72000 (CINVF), and At4g09510 (CINV2) was confirmed: GFP-containing proteins of sizes expected for the CINV–GFP fusions were present in infiltrated leaves as expected and absent from control leaves, and GFP fluorescence in infiltrated leaves was confined to the cytosol (Supplementary Fig. S2). Transcript levels in roots for these genes [from publicly available data (www.bar.utoronto.ca; Dinneny *et al.*, 2008)] are substantially lower than that for *CINV1* (~5-fold or more; Supplementary Table S2), and individual T-DNA insertion mutants for CINVB, D, and F each had near-normal root growth (Supplementary Fig. S2). However given that loss of CINV1 and CINV2 together has a stronger effect on growth than loss of CINV1 alone (Lou *et al.*, 2007; Qi *et al.*, 2007; Barratt *et al.*, 2009), future generation and characterization of lines lacking other combinations of CINVs is warranted. Further work in this study was confined to the *cinv1 cinv2* mutant.

### Root growth defects in the *cinv1 cinv2* mutant are partially restored by exogenous sugars

The roots of *cinv1 cinv2* mutants had smaller root meristems (i.e. fewer epidermal cells between the QC and the meristem–elongation zone boundary), and shorter mature epidermal cells than those of wild-type plants (Fig. 1C–E; Supplementary Fig. S2). Soil-grown *cinv1 cinv2* plants had much smaller leaves, smaller leaf cells, and a higher density of stomata than wild-type plants (Supplementary Fig. S3; Supplementary Table S3).

Previous work showed that exogenous glucose (55 mM or 30 mM) or sucrose (30 mM) partially restored root growth in the *cinv1 cinv2* mutant (Barratt *et al.*, 2009; Barnes and Anderson, 2018). We confirmed the growth-promoting effects of exogenous hexoses, and showed that neither mannitol (a control for osmotic effects; see Osuna *et al.*, 2007) nor the non-metabolizable glucose analogue 3-*O*-methylglucose had these effects (Fig. 1F). Provision of near-saturating concentrations of exogenous sugar did not restore root length in *cinv1 cinv2* mutants to wild-type values (Fig. 1F; Barratt *et al.*, 2009; Barnes and Anderson, 2018), but the glucose sensitivity of root growth was almost identical in wild-type and mutant roots. In other words, the relationship between glucose concentration and growth enhancement was the same in the two genotypes (Fig. 1G).

As well as promoting elongation, exogenous glucose increased root meristem size and epidermal cell length. Differences in epidermal cell length between wild-type and mutant roots were eliminated by exogenous glucose, and differences in meristem size were reduced (Fig. 1D, E; Supplementary Fig. S2).

### Root meristem organization is defective in *cinv1 cinv2* mutants

**Fig. 2.**
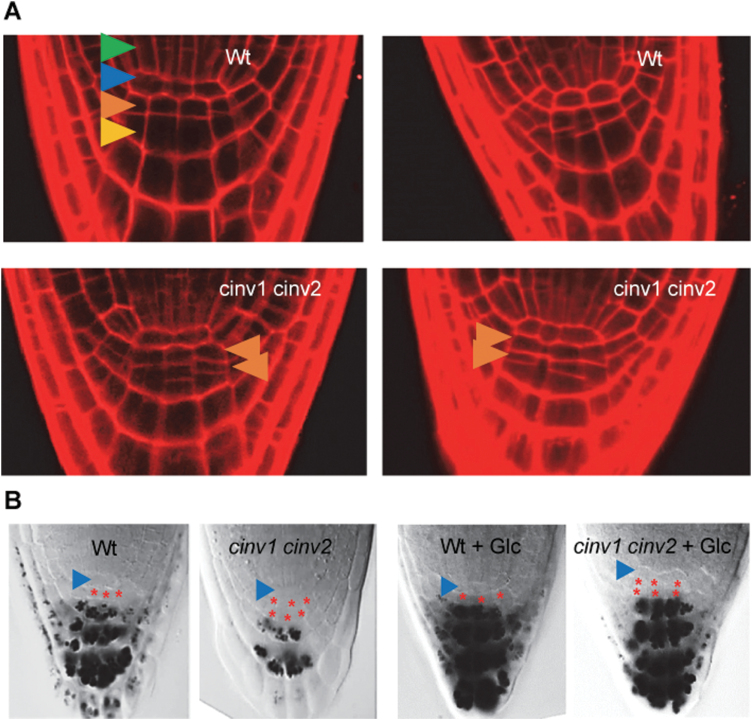
Developmental abnormalities in roots of the *cinv1 cinv2* mutant. (A) Overview of cell positions and cell fate in the root meristem. Arrows indicate cell types as follows: green, stele initials; blue, quiescent centre; orange, columella stem cells in D1 position; yellow, columella differentiated cells in D2 position. Two examples each of wild-type (upper panel) and *cinv1 cinv2* (lower panel) roots from 4-day-old plants are shown. (B) Differentiation status of columella cells in 4-day-old seedlings. Roots were stained with propidium iodide. Arrows indicate columella stem cells.

Wild-type root meristems typically have one layer of starchless columella stem cells distal to the QC and about three layers of differentiated, starch-containing columella cells between the columella stem cells and the root cap. In *cinv1 cinv2* roots, a second layer of columella stem cells was usually present (Fig. 2). Only 9% of wild-type roots possessed a second stem cell layer, whereas it was observed in 91% of mutant roots (values from 80–100 root tips). Exogenous glucose (55 mM or 220 mM) did not restore wild-type meristem organization in *cinv1 cinv2* mutants (Fig. 2B).

**Fig. 3.**
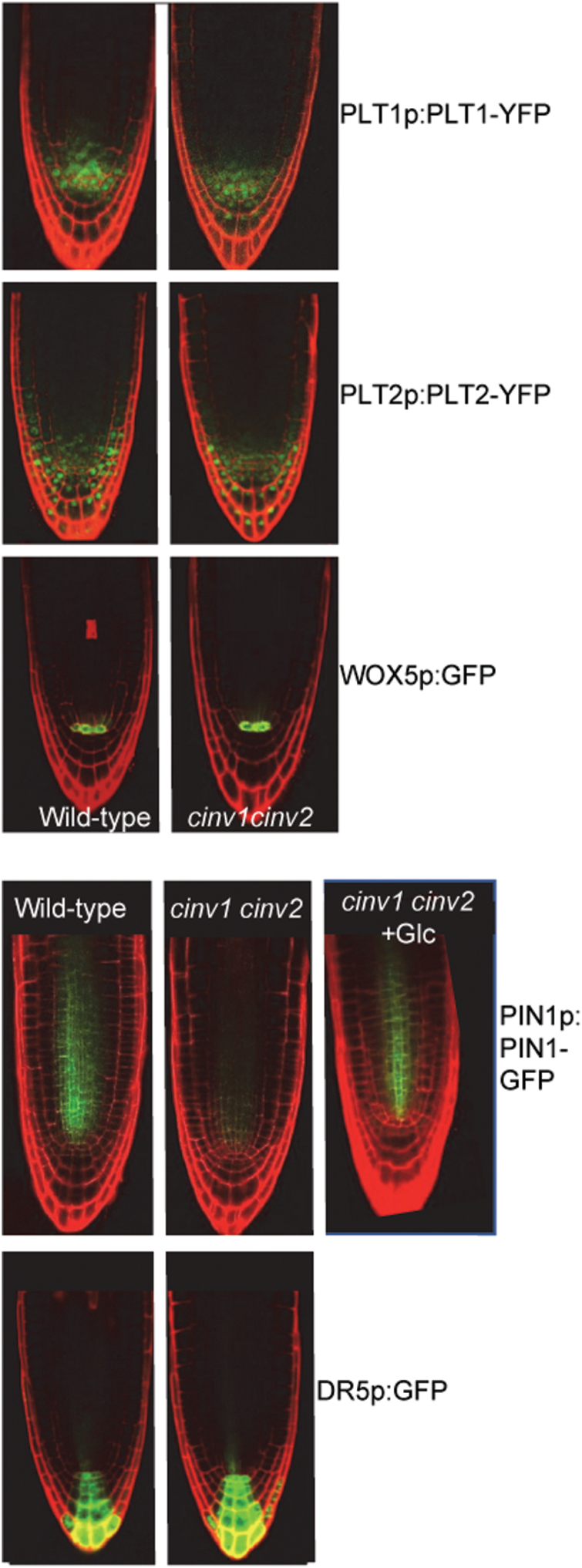
Expression of root meristem markers in *cinv1 cinv2* roots. Confocal images of 4-day-old roots stained with propidium iodide. Expression of PIN1–GFP is lower in *cinv1 cinv2* than in wild-type roots in the absence of glucose, but similar when 55 mM glucose is exogenously supplied. Expression of DR5:GFP, WOX5:GFP, PLT1:PLT1–YFP, and PLT2:PLT2–YFP is similar in *cinv1 cinv2* and wild-type roots.

To explore this defect further, we examined whether the location of transcripts for some ‘master regulators’ of meristem patterning and QC specification was perturbed in *cinv1 cinv2* mutants. Introduction of *cinv* mutations into appropriate reporter lines did not alter the locations of transcripts for *PLETHORA* (*PLT1* and *PLT2*) and *WOX5* transcription factors, which are essential for normal patterns of cell division in the columella (Fig. 3; Aida *et al.*, 2004; Galinha *et al.*, 2007; Sarkar *et al.*, 2007; Shimotohno *et al.*, 2018). Transcriptome analysis (see below; Supplementary Tables S5–S7) showed that the level of *WOX5* transcript was elevated in roots of the *cinv1 cinv2* mutant with and without exogenous glucose (log2-fold difference of 1.34 and 1.44 with and without glucose, respectively). *WOX5* overexpression gives rise to additional layers of columella stem cells (Sarkar *et al.*, 2007), hence it might cause the additional layer in the *cinv1 cinv2* mutant. However there was no change or a decrease in transcript levels in the mutant for *PLT1*, *PLT2*, *PLT3*, and *SCARECROW* (see below; Supplementary Tables S5–S7), encoding transcription factors which are implicated in induction of *WOX5* expression *in vivo* (Shimotohno *et al.*, 2018).

Since auxin gradients are also important determinants of patterns of meristematic cell division (Blilou *et al.*, 2005), we checked whether the auxin level or distribution was perturbed in *cinv1 cinv2* roots. The mutations did not affect the location of PIN1, an auxin transporter essential for establishment of auxin gradients. However the PIN1–GFP signal was markedly lower in *cinv1 cinv2* mutant roots than in wild-type roots in the absence of glucose. The signal was restored when plants were grown in the presence of glucose (Fig. 3). Consistent with these observations, transcriptome analysis (see below; Supplementary Tables S5–S7) showed that *PIN1* transcript levels were lower in mutant than in wild-type roots in the absence of glucose, but not different when glucose was supplied. Mutant roots also had an apparently normal pattern of auxin distribution (visualized through introduction of the *GFP* gene driven by the DR5 promoter: Fig. 3; Ulmasov *et al.*, 1997). Finally, growth of mutant seedlings in the presence of the synthetic auxin 1-naphthaleneacetic acid did not restore wild-type root elongation or meristem organization (Supplementary Fig. S4). Thus it seems unlikely that altered auxin levels or distribution patterns are responsible for the root elongation and meristem organization defects in the *cinv1 cinv2* mutant.

### Wild-type and *cinv1 cinv2* roots have different levels of primary metabolites

We compared levels of primary metabolites in roots of wild-type and *cinv1 cinv2* seedlings grown on agar for 4 d or 7 d. Results were similar at these two stages (Table 1). Sucrose levels were higher (by 3- to 5-fold) in mutant than in wild-type roots. Consistent with the established link between levels of sucrose and the signalling metabolite trehalose 6-phosphate (Tre6P) (Lunn *et al.*, 2006; Yadav *et al.*, 2014), mutant roots had 3–5 times more Tre6P than wild-type roots. As reported for whole seedlings by Barnes and Anderson (2018), glucose and fructose levels were also higher in mutant than in wild-type roots (10-fold and 4- to 6-fold, respectively; Table 1). Fructose levels were substantially lower than those of glucose. Despite these high sucrose and hexose levels, levels of hexose phosphates and glycolytic intermediates were 2- to 3-fold lower in mutant than in wild-type roots. As reported by Barnes and Anderson (2018), UDP-glucose levels were also lower—by almost 2-fold—in mutant than in wild-type roots. Similar trends were seen in shoots of older, soil-grown plants (Supplementary Table S4).

**Table 1.** Metabolite contents of wild-type and mutant roots

	Four days		Seven days	
	Wild type	*cinv1 cinv2*	Wild type	*cinv1 cinv2*
	μmol g^-1^ FW			
Glucose	0.24±0.12	2.20±0.65*	0.43±0.16	4.39±1.13*
Fructose	0.17±0.11	0.96±0.26*	0.13±0.09	0.57±0.24*
Sucrose	0.49±0.12	2.23±0.32*	0.49±0.27	1.56±0.21*
	nmol g^–1^ FW			
Starch	ND	ND	76 ± 11	45 ± 19
Trehalose 6P	0.011±0.003	0.055±0.014*	0.029±0.003	0.107±0.022*
Glucose 6P	30.2±8.3	14.3±0.5*	49.3±5.6	34.7±8.4
Glucose 1P	3.65±0.38	1.30±0.08*	11.43±0.61	4.47±0.08*
Fructose 6P	10.2±2.7	5.0±0.5*	35.9±1.8	14.1±3.1*
UDP-glucose	20.8±2.4	11.0± 3.7*	50.8±3.1	28.6±5.3*
3-PGA	7.1±1.8	3.4±0.7*	49.8±6.4	21.0±3.1*
PEP	3.9±1.1	1.1±0.2*	10.1±1.5*	4.8±0.5*

Roots were from 4- or 7-day-old seedlings grown on vertical agar plates without sugar. Values are the means ±SD of measurements on three samples, each of 30–40 roots. Student’s *t*-test (two-tailed, equal variance) was used to test the significance of differences between mutant and wild-type values. *P<0.05. ND, not determined.

Elevated hexose levels in *cinv1 cinv2* mutant plants were not associated with general increases in transcripts for other sucrose catabolic enzymes. For roots of 4-day-old seedlings (analysed by RNA-seq; see next section; Supplementary Table S5), transcript levels for *A/N-InvB*, *A/N-InvC*, *A/N-InvD*, *A/N-InvH*, a vacuolar INV At1g12240, and the sucrose synthases *SUS2* and *SUS4* were reduced in mutant relative to wild-type roots, and transcript levels for phloem-located sucrose synthase *SUS6* (Barratt *et al.*, 2009) and a cell wall acid invertase *cwINV1* were elevated. For 14-day-old seedlings, there was modest elevation of transcript levels for *A/N-InvB*, *A/N-InvD*, and *SUS4* (qPCR analysis; Supplementary Fig. S1).

### Transcript levels for thousands of genes are altered in *cinv1 cinv2* seedlings

The above results indicated that the phenotype of *cinv1 cinv2* roots is determined by several distinct phenomena. Although mutant roots had high levels of sucrose, Tre6P, and hexoses, two observations point to the occurrence of low carbon availability for metabolism: (i) the reduced levels of hexose phosphates, UDP-glucose, and glycolytic intermediates; and (ii) the full or partial restoration of some aspects of root growth by exogenous hexoses. However, the fact that exogenous hexoses cannot fully complement the mutant phenotype argues against low carbon availability as the sole explanation for the *cinv1 cinv2* phenotype. To shed light on the metabolic and developmental networks compromised in the *cinv1 cinv2* mutant, we analysed the transcriptome of roots of 4-day-old seedlings grown with and without 55 mM glucose. At this early stage, seedlings of the two genotypes are similar in size and morphology, hence pleiotropic effects due to different growth rates will be relatively small (Supplementary Fig. S1). Validation and evidence of the reproducibility of RNA-seq data are shown in Supplementary Fig. S1. Results described below encompass all genes for which differences in transcript levels between mutant and wild-type plants were statistically significant (see the Materials and methods); a fold change cut-off has not been applied. Note that qPCR analysis confirmed the direction of change and largely confirmed the magnitude of difference for 38 transcripts, most of which gave log2-fold changes of <2 (Supplementary Fig. S1). We first describe genes that were differentially expressed between wild-type and mutant roots [differentially expressed genes (DEGs)] in the presence or absence of exogenous glucose, and then the different effects of exogenous glucose on the transcriptomes of the two genotypes.

**Fig. 4.**
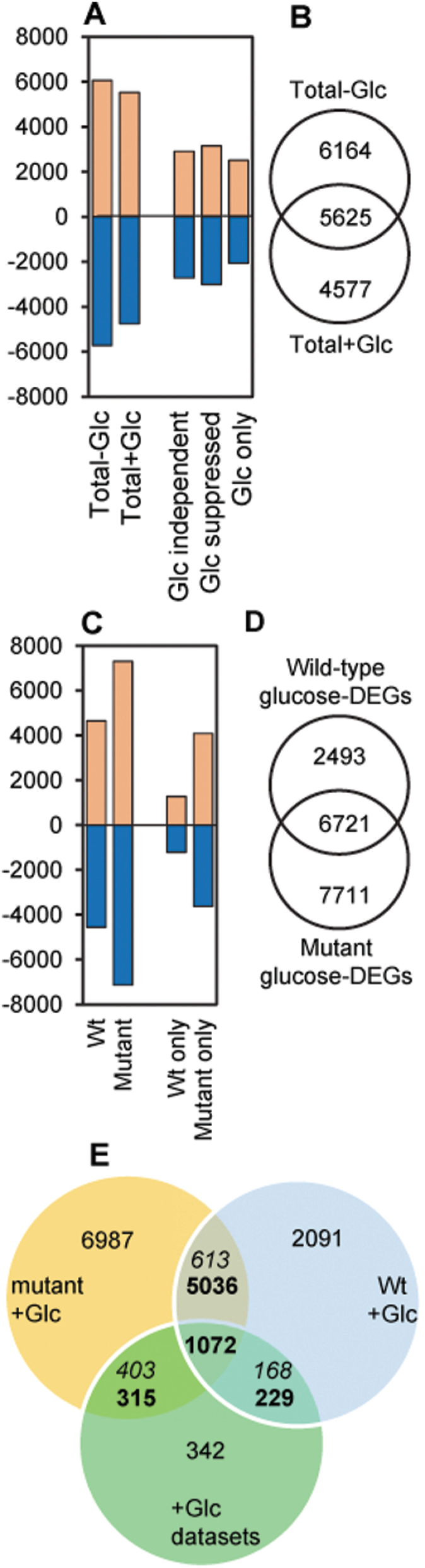
Differences in transcript levels between 4-day-old wild-type and *cinv1 cinv2* roots. (A) Left: numbers of differentially expressed genes (DEGs) in the absence of glucose in the growth medium (Total–Glc) and in the presence of glucose in the growth medium (Total+Glc), and estimates from these data of numbers of genes differentially expressed whether or not glucose was added (Glc independent), number differentially expressed only in the absence of glucose (Glc suppressed), and number differentially expressed only in the presence of glucose (Glc only). Orange bars: numbers of genes expressed at a higher level in mutant than in wild-type roots. Blue bars: numbers of genes expressed at a lower level in mutant than in wild-type roots. (B) Degree of overlap of individual DEGs in the –Glc and +Glc datasets; (C) Numbers of genes differentially expressed in the presence versus the absence of glucose (glucose-DEGs). Left, glucose-DEGs in wild-type and mutant roots; right, numbers of individual glucose-DEGs exclusive to wild-type or mutant roots (Wt only and mutant only, respectively); colours are as for (A). (D) Degree of overlap of individual glucose-DEGs in the wild-type and mutant datasets. (E) Degree of overlap of the wild-type and mutant glucose-DEGs with each other and with a set of previously identified glucose-responsive genes (+Glc datasets). In overlaps, bold type indicates the number of genes that changed in the same direction between two datasets, and italics indicates the number of genes that changed in opposite directions.

Despite the similarity in appearance of wild-type and mutant seedlings at 4 d, an exceptionally large number of transcripts accumulated to different levels in roots of the two genotypes (Fig. 4A; Supplementary Tables S5–S7). In the absence of exogenous glucose, there were nearly 12 000 DEGs. Provision of 55 mM exogenous glucose reduced total DEGs by only 13% (Fig. 4A, B). However, exogenous glucose had a profound effect on which genes were represented in the DEG set. About 6200 individual genes that were differentially expressed in the absence of exogenous glucose were no longer differentially expressed when glucose was supplied. A further 5600 individual genes were differentially expressed whether or not glucose was supplied (referred to as Glc-independent DEGs: Fig. 4A, B; Supplementary Table S7), and another 4600 individual genes were differentially expressed in the two genotypes only when glucose was supplied (Glc only DEGs: Fig. 4A).

DEGs encompassed many, very diverse cellular processes. In the absence of exogenous glucose (Total–Glc), GO categories in which markedly more DEGs were up-regulated than down-regulated included ‘response to stress’ and ‘transcription, DNA dependent’. Categories in which more DEGs were down-regulated than up-regulated included ‘protein metabolism’, ‘transport’, ‘cell organization and biogenesis’, ‘developmental processes’, and ‘electron transport or energy pathways’ (Supplementary Figs S4, S5). Down-regulated genes in the cell organization category included those encoding expansins, cell wall proteins, and ribosomal proteins. Somewhat similar trends were present in glucose-independent DEGs: the majority of genes in the ‘response to stress’, ‘signal transduction’, and ‘transcription, DNA dependent’ GO categories were up-regulated, whereas the majority in the categories ‘protein metabolism’, ‘cell organization and biogenesis (including cell wall proteins)’, ‘transport’, and ‘electron transport or energy pathways’ were down-regulated (Supplementary Fig. S5). In total, >400 genes annotated as transcription factors were up-regulated in mutant relative to wild-type roots in a glucose-independent manner. Many genes involved in hormone metabolism and perception, calcium signalling, and signal perception (receptor kinases) were also differentially expressed between mutant and wild-type roots (Supplementary Fig. S6). Transcript levels for most genes in the mitochondrial and plastid genomes were lower in mutant than in wild-type roots, including genes related to translation in these organelles (Supplementary Tables S5, S6). In broad terms, these differences indicate a general increase in stress perception and response, and a decrease in processes related to cell structure, maintenance, and growth in mutant relative to wild-type seedlings, regardless of whether glucose was supplied.

Given the high levels of both glucose and sucrose in *cinv1 cinv2* relative to wild-type roots, we examined whether genes that were differentially expressed between the two genotypes are known from previous studies to be responsive to manipulation of glucose and/or sucrose levels in seedlings. It is important to note that previous studies differed from each other (and from our experiments) with respect to numerous factors including growth stages and conditions, and the timing and concentration of exogenous sugar applications, and with respect to the number and nature of genes reported as sugar responsive. Nonetheless, these studies collectively allowed us to compile lists of sucrose- and glucose-responsive genes with which DEGs in our study could be compared. We used five widely cited studies of transcriptional responses to exogenous sugars (Price *et al.*, 2004; Bläsing *et al.*, 2005; Gonzali *et al.*, 2006; Li *et al.*, 2006; Osuna *et al.*, 2007) (Supplementary Tables S8, S9). In the absence of glucose (Total–Glc), ~1500 of the 12 000 individual DEGs were identified as glucose-responsive genes in the previous studies (Supplementary Table S10). However, only half of the 1500 genes differed in expression between mutant and wild-type roots in the direction expected if glucose levels were elevated in mutant relative to wild-type roots. For the other half, differences were in the direction expected if glucose levels were reduced in mutant roots (Supplementary Table S10).

The glucose-independent DEGs might be expected to be sucrose-responsive genes, and indeed over two-thirds of these individual genes (~4000 genes) were reported to be sucrose regulated in previous studies (Supplementary Table S11). However, as for glucose-responsive genes, only half of the 4000 genes differed in expression between mutant and wild-type roots in the direction expected if sucrose levels were elevated in mutant relative to wild-type roots. The other half differed in expression in the direction expected if sucrose was lower in mutant than in wild-type roots.

### Exogenous glucose has different effects on the transcriptomes of mutant and wild-type roots

The transcriptomes of wild-type and *cinv1 cinv2* roots differed profoundly in their response to exogenous glucose, in terms of both total numbers and individual genes involved. About 9200 genes responded to exogenous glucose (glucose-DEGs) in wild-type roots, but >14 400 genes responded in mutant roots (Fig. 4C). Of the individual glucose-DEGs in wild-type roots, 73% were also glucose-DEGs in mutant roots, and 27% were not differentially expressed in response to glucose in mutant roots. Conversely, over half of the individual glucose-DEGs in the mutant roots were not differentially expressed in response to glucose in wild-type roots (Fig. 4D, E). Among the ~6700 glucose-DEGs that were shared by wild-type and mutant seedlings, most (91%) changed in the same direction in response to glucose in the two genotypes. Expression of the remaining 9% of genes was elevated by glucose in one genotype but repressed in the other (Fig. 4E).

Glucose-DEGs for wild-type and mutant roots shared ~1470 and 1790 genes, respectively, with our list of previously identified glucose-responsive genes. In both cases, the expression of most of the glucose-DEGs changed in the direction expected from the previous studies (88% in wild-type and 78% in mutant seedlings).

In summary, the mutant transcriptome was much more responsive to exogenous glucose than the wild-type transcriptome, and more than half of the transcripts that responded to exogenous glucose in the mutant did not respond in wild-type seedlings. Nonetheless, most of the glucose-DEGs that were in common between wild-type and mutant seedlings showed the same direction of response in the two genotypes (e.g. up-regulated in both or down-regulated in both) and in the list of genes previously identified as glucose-responsive genes.

## Discussion

### Impact of reduced CINV activity on sucrose and hexose levels

Because CINV activity is central to the entry of imported sucrose into metabolism in Arabidopsis roots, large reductions in CINV activity might be expected to increase levels of the invertase substrate sucrose, and to decrease levels of the products glucose and fructose. This expectation is borne out by our data with respect to levels of sucrose. Roots of the *cinv1 cinv2* mutant had 3–4.5 times more sucrose than wild-type roots, and sucrose was also elevated in shoots of 35-day-old mutant plants. However, rather than the expected reductions in hexose levels, there were substantial elevations in mutant plants (up to 9-fold in roots and 3-fold in shoots) (Table 1; Supplementary Table S4). Our results are broadly similar to those of Barnes and Anderson (2018), who reported elevated levels of hexoses in mutant relative to wild-type plants for 3- and 6-day-old etiolated seedlings and 3-day-old roots plus hypocotyls, and elevated sucrose for 6-day-old etiolated seedlings and 3-day-old roots plus hypocotyls. The differences in sugar levels between mutant and wild-type plants were much smaller than in our experiments (≤2-fold), but plant ages, plant parts, and growth conditions all differed from those in our experiments.

We concur with the suggestion of Barnes and Anderson (2018) that the unexpected elevation of hexoses in mutant roots is due to hydrolysis of sucrose in a cellular compartment other than the cytosol, and retention of the resulting hexoses in that compartment. The capacity for sucrose hydrolysis in the cytosol is strongly decreased in the mutant (Barratt *et al.*, 2009), but isoforms of invertase also occur in cell walls and vacuoles (acid invertases) and in mitochondria and plastids (organelle-specific isoforms of neutral invertase). We found no evidence for major elevation of transcripts encoding acid and organellar invertases in *cinv1 cinv2* (Supplementary Fig. S1A), but this does not rule out the possibility that accumulation of high concentrations of sucrose in the cytosol increases its concentration in other invertase-containing cellular compartments (e.g. vacuoles), leading to enhanced sucrose hydrolysis and hence elevated glucose specifically in these compartments rather than in the cytosol. Some features of the *cinv1 cinv2* phenotype are consistent with the idea that glucose concentrations may be low in the cytosol. These features include the low levels of intermediates of primary metabolism, the response to exogenous glucose of some aspects of root growth, and patterns of transcript abundance indicative of both high sucrose and low glucose signalling (see below).

Recent research on the relationship between sucrose import into the vacuole and the accumulation of monosaccharides in leaves lends some support to the idea that the high glucose concentrations in *cinv1 cinv2* leaves may be generated by sucrose hydrolysis in the vacuole. Increasing the transport of sucrose into vacuoles (through heterologous expression of the vacuolar sucrose loader TST2.1 from sugar beet) resulted in increased levels of monosaccharides, due to sucrose hydrolysis by vacuolar invertases (Vu *et al.*, 2020). We also considered whether altered expression of genes encoding proteins involved in movement of sugars across the tonoplast and its hydrolysis within the vacuole (Martinoia, 2018; Vu *et al.*, 2020) might contribute to glucose accumulation in the *cinv1 cinv2* mutant. No consistent picture emerged. Transcripts for two genes encoding transporters responsible for sugar entry into the vacuole—*Tonoplast Sugar Transporter1* (*TST1*, syn. *TMT1*; Wormit *et al.*, 2006) and *Vacuolar Glucose Transporter* (*VGT1*; Aluri and Büttner, 2007)—were reduced in the mutant, but this was also true of the transcript for the major tonoplast glucose exporter *Early Responsive to Dehydration-Like6* (*ERDL6*; Poschet *et al.*, 2011) (Supplementary Table S5). The transcript for one of the two vacuolar acid invertases, *VI2*, was also reduced, but transcript levels of *VI1*, the other vacuolar invertase (Weiszmann *et al.*, 2018), and two further proteins implicated in sugar export (*SWEET16* and *SUC4*; Endler *et al.*, 2006; Klemens *et al.*, 2013) were unaffected (Supplementary Table S5). Further research is required to establish conclusively the reasons for and location of the high glucose contents of *cinv1 cinv2* seedling roots, taking into account that both tonoplast sugar transporters and vacuolar invertase activity are regulated at post-translational as well as transcriptional levels (e.g. Wingenter *et al.*, 2011; Vu *et al.*, 2020).

### *cinv1 cinv2* mutants exhibit symptoms of carbon starvation

Two main features of the *cinv1 cinv2* phenotype are indicative of low availability of substrate for primary metabolism, consistent with the idea that glucose concentration in the cytosol may be low. First, levels of starch and glycolytic intermediates (hexose phosphates, 3-phosphoglycerate, and phosphoenolpyruvate) in the roots of mutant plants were significantly lower than those in wild-type plants. At 4 d, levels of all the intermediates in mutant roots were about half or less of wild-type values, indicating that glycolytic flux may be low in mutant plants. Levels of UDP-glucose, the substrate for cellulose synthesis, were also substantially lower in mutant than in wild-type roots. Barnes *et al.* (2018) reported similarly reduced levels of UDP-glucose in 3-day-old *cinv1 cinv2* seedlings, and suggested that this might be directly responsible for the 18% reduction in crystalline cellulose content they observed in mutant relative to wild-type seedlings. Rende *et al.* (2017) reported that transgenic poplars with reductions of up to 60% in neutral invertase activity in developing wood had UDP-glucose levels up to 50% lower than those of wild-type plants, and reductions of up to 13% in crystalline cellulose content. These findings suggest that neutral invertase is important for the conversion of sucrose to UDP-glucose and hence for cellulose synthesis.

In general, the source of UDP-glucose for cellulose synthesis remains unclear. It can be generated directly from sucrose via SUS, and models to explain the mechanism of cellulose synthesis often show UDP-glucose synthesis via SUS. Doubt has been cast on this idea by the demonstration that (i) plants deficient in or lacking sucrose synthase may display no specific reduction in cellulose content [e.g. an Arabidopsis mutant lacking all four soluble SUSs (Barratt *et al.*, 2009), and transgenic alfalfa and hybrid aspen with strong reductions of SUS in their stems (Gerber *et al.*, 2014; Samac *et al.*, 2015)]; (ii) SUSs are almost exclusively associated with the phloem in adult plants and not with cell types in which cellulose synthesis is occurring (Yao *et al.*, 2020); and (iii) reductions in CINV can reduce both UDP-glucose and crystalline cellulose levels (see above). These findings are consistent with a role for neutral invertase in conversion of sucrose to UDP-glucose for cellulose synthesis, but they do not rule out the possibility that both SUS and neutral invertase may contribute to the pool of UDP-glucose accessed by cellulose synthases in some locations and circumstances.

The second feature of the *cinv1cinv2* phenotype indicative of low carbon availability is the response of growth of the mutant to exogenous provision of glucose. Glucose restored root meristem size and the length of cells in the elongation zone to near-wild-type levels, suggesting that some specific aspects of root growth were carbon limited in the mutant. However, other aspects of growth were not restored by exogenous glucose. Promotion of root elongation by glucose was the same in mutant and wild-type roots, hence the restricted elongation in mutant relative to wild-type roots was not due to lower carbon availability in the mutant (Figs 1, 2).

### The *cinv1 cinv2* phenotype is not simply due to reduced supply of metabolic substrates

Although some of the effects of reduced CINV probably stem from low availability of substrates for primary metabolism and growth, this is unlikely to be the sole explanation for the profound defects in the *cinv1 cinv2* mutant. First, mutant roots retain a considerable capacity for sucrose catabolism. They have 40–50% of wild-type neutral invertase activity (Barratt *et al.*, 2009), and near-normal transcript levels for numerous other enzymes of sucrose catabolism. Second, exogenous glucose failed to correct several of the developmental defects in the mutant. Although it promoted cell elongation and meristem size in the mutant, it did not correct the defects in root elongation and in cell division below the QC(Figs 1, 2). Thus many of the defects in mutant plants may stem from signals generated by altered sucrose and hexose levels in the cytosol, rather than directly from low carbon availability for primary metabolism and growth. Below we discuss the nature of these signals and how they may impact growth and developmental processes.

### Disruption of auxin signalling does not explain the *cinv1 cinv2* phenotype

Sugar signals are known to influence the transport and activity of auxin, which plays a central role in root architecture and growth. The establishment of auxin concentration gradients and local maxima is essential for meristem maintenance and correct patterns of cell proliferation and differentiation. Mutants with defective auxin synthesis, transport, or perception display developmental abnormalities including reduced and disorganized root meristems (e.g. Blilou *et al.*, 2005; Dharmasiri *et al.*, 2005; Ding and Friml, 2010). There are numerous examples of modulation of auxin action by sugar levels. For example, root meristem size was reduced by exogenous sucrose and high levels of glucose (>150 mM) because expression of the auxin transporter PIN1 was repressed and hence auxin levels were reduced (Yuan *et al.*, 2014). In seedlings, exogenous glucose and sucrose both promoted auxin synthesis (Sairanen *et al.*, 2012), and exogenous glucose modulated the effects of exogenous auxin on gene expression (Mishra *et al.*, 2009). We therefore considered whether the effects of altered sugar levels in *cinv1 cinv2* on root development and gene expression might stem from altered patterns and/or levels of auxin.

Although transcript levels for some genes implicated in auxin signalling and downstream actions were altered in the *cinv1 cinv2* mutant, our results suggest that disruption of auxin homeostasis is not the primary cause of growth and developmental defects in the mutant. *PIN1* was differentially expressed in the absence of glucose, but its expression was the same in wild-type and mutant plants when glucose was supplied. There were no major changes in the level or pattern of expression of *PLT* and *WOX5*, transcription factor genes downstream of auxin that are crucial determinants of cell fate in the root meristem (Fig. 3) (Shimotohno *et al.*, 2018). These observations do not rule out a role for auxin signalling in some aspects of the *cinv1 cinv2* phenotype. For example, two (or more) layers of columella stem cells (rather than a single layer as in wild-type roots) are present in auxin biosynthesis and transport mutants in which the auxin maximum below the QC is disrupted (Friml *et al.*, 2002; Ding and Friml, 2010).

Numerous other differences between *cinv1 cinv2* and wild-type plants may also contribute to the root growth and developmental defects of the mutant. For example, expression of many genes involved in meristem maintenance and cell fate determination in roots is altered in the *cinv1 cinv2* mutant, including genes encoding components of the cyclin D/retinoblastoma/E2F pathway that controls the cell cycle (Cruz-Ramirez *et al.*, 2013; Desvoyes *et al.*, 2014), and levels of the sucrose signalling metabolite Tre6P (discussed below) are strongly elevated in the mutant (Table 1). Tre6P is implicated in development and meristem identity in several plant systems (Eastmond *et al.*, 2002; Satoh-Nagasawa *et al.*, 2006; Eveland and Jackson, 2012; Wahl *et al.*, 2013; Yadav *et al.*, 2014; Claeys *et al.*, 2019).

### Reciprocal changes in cytosolic sucrose and hexose levels have complex implications for sugar signalling

As discussed above, it seems likely that many features of the phenotype of the *cinv1 cinv2* mutant stem from perturbed sugar signalling. Two protein kinase complexes with apparently opposing functions have been widely implicated in the perception and transduction of sugar signals in plant cells. The SNF1-related protein kinase 1 (SnRK1) complex is activated when sugar status is low and is inhibited by high sugar status. In particular, it is inhibited by hexose phosphates and by Tre6P (Baena-González and Lunn, 2020). The target of rapamycin (TOR) complex is activated when sugar status is high and acts to promote biosynthetic and growth processes. For example, provision of glucose to carbon-starved Arabidopsis seedlings reactivated growth in quiescent root meristems in a TOR- and glycolysis-dependent manner (Xiong *et al.*, 2013; Baena-González and Hanson, 2017). Our data suggest that the cytosol of root cells in the *cinv1 cinv2* mutant has a higher concentration of sucrose but a lower concentration of glucose than that of wild-type plants. In this situation, SnRK1 activity may be determined by a balance between the inhibitory effects of the high Tre6P levels brought about by elevated sucrose, and activation by low hexose phosphate levels brought about by low glucose. TOR in mutant roots may well be inhibited by low glucose levels and apparently low rates of glycolysis. Further complications are (i) transcript levels for both SnRK1- and TOR-associated proteins were reduced in mutant relative to wild-type roots (Supplementary Table S5; reduced transcripts for SnRK1 components AKINβ1, AKIN10, and AKIN11; and TOR and its binding partners RAPTOR1 and RAPTOR2); and (ii) provision of exogenous glucose to mutant roots altered the expression of very large numbers of genes, most of which have not previously been reported to be glucose sensitive (Fig. 4; Supplementary Tables S5, S6).

From the considerations above, we suggest that the large and complex transcriptional differences between mutant and wild-type roots reflect signalling from the combination of low glucose levels and high sucrose levels in the cytosol in mutant roots. Significant numbers of genes known to respond to sucrose changed in the direction expected if sucrose levels are high, for example the mutant had reduced expression of the starvation-induced genes *DIN1*, *DIN2*, *DIN3*, *DIN6*, *DIN9*, and *DIN10*, and elevated expression of nitrate reductase and the Tre6P-synthesizing enzyme TPS1. However, of the 3700 DEGs known to respond to sucrose, about half changed in the opposite direction from that expected when sucrose levels are high (Supplementary Table S11). Similarly, some genes known to respond primarily to glucose changed in the direction expected if glucose levels are low, for example the mutant had elevated expression of genes associated with senescence and autophagy and reduced expression of genes associated with translation and cell organization (Supplementary Table S10). However, 40% of DEGs known to respond to glucose changed in mutant relative to wild-type roots in the opposite direction from that expected for low glucose levels.

A picture emerges from our data of a network of sugar signals that responds to variation in the ratio of sucrose to glucose and intersects with a wide spectrum of responses of gene expression. Expression of some genes responds primarily to sucrose signals or to glucose signals, but the level of expression of many genes may be set by the sucrose to glucose ratio rather than absolute concentrations of one or the other sugar. Although the *cinv1 cinv2* mutant represents a rather extreme change in sucrose to glucose ratios relative to wild-type plants, reciprocal changes in these sugars may be commonplace during normal metabolism. Root CINV activity is modulated by 14-3-3-protein-dependent phosphorylation in response to shoot illumination (Gao *et al.*, 2014), suggesting that modulation of root CINV activity may contribute to coordination of sugar supply, metabolic demand, and downstream growth and developmental processes influenced by sugar signalling over the diel cycle.

## Supplementary data

The following supplementary data are available at *JXB* online.

Table S1. Primers used in this study.

Table S2. Transcript levels for cytosolic invertases in roots.

Table S3. Leaf area and stomatal density in wild-type and mutant shoots.

Table S4. Metabolite contents of wild-type and mutant shoots.

Table S5. Transcripts differentially accumulating in the *cinv1 cinv2* mutant in the absence of glucose.

Table S6. Transcripts differentially accumulating in the *cinv1 cinv2* mutant in the presence of 55 mM glucose.

Table S7. Transcripts differentially accumulating in the *cinv1 cinv2* mutant in the presence and absence of glucose.

Table S8. Transcripts differentially accumulating in response to glucose in previous studies.

Table S9. Transcripts differentially accumulating in response to sucrose in previous studies.

Table S10. Transcripts differentially accumulating in the *cinv1 cinv2* mutant in the absence of glucose that were previously reported to be responsive to glucose.

Table S11. Transcripts differentially accumulating in the *cinv1 cinv2* mutant with and without glucose that were previously reported to be responsive to sucrose.

Fig. S1. Transcript levels for sucrose metabolizing enzymes in *cinv1 cinv2* roots, and validation of RNA-seq data.

Fig. S2. Original micrographs used in Fig. 1C.

Fig. S3. Characterization of Arabidopsis neutral invertase.

Fig. S4. Effects of auxin on *cinv1 cinv2* root elongation and developmental abnormalities.

Fig. S5. Functional characterisation of genes differentially expressed in mutant and wild-type roots (DEGs) into GO-slim Biological Processes.

Fig. S6. MAPMAN visualization of genes differentially expressed in mutant and wild-type roots whether or not glucose was supplied.

eraa581_suppl_Supplementary_Tables_S1-S4_and_Figures_S1-S6

eraa581_suppl_Supplementary_Tables_S5-S11

## Data Availability

Data supporting the findings of this study are available within the paper and within its supplementary data. Further information may be obtained from the corresponding author, Alison M. Smith.
