# Supplementary material for: Restriction of cytosolic sucrose hydrolysis profoundly alters development, metabolism, and gene expression in Arabidopsis roots
Source: J Exp Bot. 2020 Dec 30;72(5):1850–63. doi: 10.1093/jxb/eraa581 (PMC7921298; doi:10.1093/jxb/eraa581)
Supplement: eraa581_suppl_Supplementary_Tables_S1-S4_and_Figures_S1-S6 [file eraa581_suppl_supplementary_tables_s1-s4_and_figures_s1-s6.pdf]

Restriction of cytosolic sucrose hydrolysis profoundly alters development, metabolism and gene expression in Arabidopsis roots

Cristina Pignocchi, Alexander Ivakov, Regina Feil, Martin Trick, Marilyn Pike, Trevor L. Wang, John E. Lunn, Alison M. Smith

Supplementary Data

Tables S1-S4
Figures S1-S6

Tables S5-S11 are in a separate Excel spreadsheet

Table S1. Primers used in this study.

	AGI Number		Primers
q-PCR primers:			
CINV1	AT1G35580	F	AATCGCGTCTTCACCGAGACTG
		R	AACGTACCTTCCGAGCTTACCG
CINV2	AT4G09510	F	TGGCGGATTGTTACCGGTTGTG
		R	TCAGCGTCCACAGCAATACTGG
SWEET3	AT5G53190	F	GTTCTTCCGGCCAATCAAATGGG
		R	CAACAGAGAAGCTCCGTTTCCC
SWEET11	AT3G48740	F	TTGCCGTGTTCCTATCTCCTGTGC
		R	ACTGAAGAGCGCCACAACATAAGG
SWEET12	AT5G23660	F	ATCTCCATCTTTGTTGCCTTCGC
		R	CAAGAGCTTCACCGTTAGCATTCG
GRF1	AT2G22840	F	TCGGCGAGGTACTAAACAGCAC
		R	TTGCAAGACGCCTGTTGGAGAG
GRF4	AT3G52910	F	ATCACCAACCTTCTTGGTATTGGG
		R	TGCCGTCAGTTCTCTTACACCTC
GRF6	AT2G06200	F	CTCCCACTCTTTCTCCACACTTTG
		R	TCTACATCTTCCTGGCTCTGCATC
TPPH	AT4G39770	F	GAGACTAGCGCGTCTTATTCACTG
		R	ACGTTGCAAGAACTCCATAACCTC
SUC2	AT1G22710	F	TAGCCATTGTCGTCCCTCAGATG
		R	ACCACCGAATAGTTCGTCGAATGG
NCED5	AT1G30100	F	ACGCCGTTACGTTGGAACTAGAAG
		R	TCACGAAAGTGCCGTGGAAACC
PCNA	AT1G07370	F	CGGTGACATTGGAACCGCTAAC
		R	TCACAATTGCATCTTCCGGCTTG
PIN4	AT2G01420	F	ACAACGTGGCAACGGAACAATC
		R	GCCGATATCATCACCACCACTC
RPL5	AT2G07725	F	AAAGGAGGAGTAGGAGGAGTCAGC
		R	TTGGCGTGATTCGGTTTGAGC
EXP-A	AT2G03090	F	GCATTGCTCAGTACAAAGCTGGTG
		R	CTTCTCATACACGGAACCCTTCTG
ATGLR2	AT2G24710	F	TGAGGCTGTGATTCGAGAACTGC
		R	TCCCACAACCGCATCATATCTCC
WOX5	AT3G11260	F	TTTGAAGACTCCAACTCCAAGGTG
		R	GCGGTGGATGTTCCATTTCAGC
WOX4	AT1G46480	F	ACGACCACTGGTGTCTTTAATCCG
		R	CTCCTCCTTCTCCACCATTGGTTC

PIN5	AT5G16530	F	ATGGCCATCGGCTCTATTGTCC
		R	AGCAGCCTGAATGATGGCTACG
FAF1	AT4G02810	F	TGGCCAAGCTTACCAACATCTTCC
		R	AGAAACTCCATCCACCCACGTC
FAF4	AT3G06020	F	TAATGATGGCCACGAGACCTCCTC
		R	TCTGACACAGCCATTGCTTCGG
SMP1	AT1G65660	F	AGAGGCTGCCCTTGATCTGATG
		R	TTCAACCTTCTTTGGGCTCTCTTC
SMP2	AT4G37120	F	CTCAAGTCTGAACAGCCGAGTTTG
		R	ACGCTCCCTTGCGGTATTGTTC
RGF1	AT5G60810	F	TGCAGGTGCATGCTAAAGTCTCC
		R	AGCATGCTTCTTGGCAATTTGGG
ANT	AT4G37750	F	TCAATACCGAGGCGTTACAAGAC
		R	TCGAGCAGCTTTCTCCTCCATATC
LOG2	AT2G35990	F	ATGCCTTCATCGCACTTCCTGGTG
		R	AGCTGAGACCAGGTGATGACTTCC
MAP65	AT1G14690	F	TGGTTATCTGCGTGCGAAGAGG
		R	GGCACTATACCGGGTTTCATCCTG
KAN3	AT4G17695	F	AGGGCAGTCAGATTGTGAGAATGG
		R	AACTGGAACCGAGCTTCACTTG
BEL1	AT5G41410	F	GCGGCAACAGAAATCGTATCGC
		R	ACATCGCTCGGATATGGGTGAAG
HB32	AT1G14687	F	GCGGAGGAAGTCAAAGTTCACG
		R	ATCCCAACTTCGCCGCGTAATC
HB53	AT5G66700	F	ACAGGTGGCTGTTTGGTTCCAG
		R	TCTGAGACTCGAGTTGGCATTGG
NEK5	AT3G20860	F	AGAACGAGGTTCGGTTAGGTGAC
		R	TCAGGGCACATGTAGTTTGGTG
NEK6	AT3G44200	F	CGGATGACCTAACTTCCTCGGTTG
		R	AGGGATATCAGCAAGCAGTTCCG
MATE	AT2G04070	F	TGTAGTGGTTGGATCCGCTGTG
		R	TCCTTGCCTTCTTGGCCTGTTC
CPK22	AT4G04710	F	CATCTTGCAACCACAAGGGAAAC
		R	ATGTGCCCGCTGTTGTCTTTATC
BGLU28	AT2G44460	F	TCACCTTCTTCTCGCTCATGCC
		R	CCAATCTGACCGTCTTGAGTCTTG
DOT2	AT5G16780	F	AGTGGCCATGTCAAACCAGGAC
		R	TCCTTCTCAACAGTCGCAAACCC
SUS6	AT1G73370	F	AGTCCTTCGTCAATGGGTTTCCC
		R	AATCTTGCTCGTTGCGTCCTGAG
HSP70	AT3G12580	F	CTACCAACACCGTCTTCGATGC
		R	GACTCTTATCCGCTTGAACAGAGG

NAC071	AT4G17980	F	AGGTTGCTTCTGAGTGCTTTCCC
		R	AAATTCCACGTGGTGGTTTGCC
INVD	AT1G22650	F	GCTGCATGCATAAAGACGGG
		R	GATTTCCCGTCGTAGTACTCCG
INVH	AT3G05820	F	GGTCTTACCACAATGGTGGGTC
		R	GAGCCTCTTCTCTGCCAAGGTT
INVC	AT3G06500	F	CCAACTCTCCTCTGGCAGTTCA
		R	GGCCACTCATCTTCCTTTAGCC
INVB	AT4G34860	F	AATGGAGGATCGTGGCCAGT
		R	CCTTATGAAGCCTAGCCTCTGC
INVA	AT1G56560	F	TGGGAAGACCAGAGCTAGCTGA
		R	GGCGAGACTGCTTTCCAATG
INVE	AT5G22510	F	CAACTCTGCTATGGCAGCTGAC
		R	CGTAGTACTCGGGCCATTTGTC
CWINV1	AT3G13790	F	GTTGTGCTCATGTGCAGTGACC
		R	TGAGGGATAGTGGTTGGTGAGG
CWINV6	AT5G11920	F	GTTATTCGTCGCGGTAACGG
		R	AGGAATGCTCCATGCGAAGA
VINV2	AT1G12240	F	GAAGCATTCGGACAAGGTGG
		R	CCGTAACCGTCGCATCAAGA
VINV1	AT1G62660	F	GAAGGATTCGCACAAGGTGG
		R	GTAACGGTCGCATCAATGGC
18S	AT2G01010	F	GGTACGTGCTACTCGGATAACC
		R	TCTCCGGAATCGAACCCTA
SUS1	AT5G20830	F	TACCGACTTTCGCCACTTGC
		R	TCAGCAGCCTGATCACCATG
SUS2	AT5G49190	F	TGACTTGTGCACTCCCAACG
		R	CCTGGTCTGGATGATATGGGTC
SUS3	AT4G02280	F	CGAGGCTTTTGGACTTACGG
		R	TCGATGTGGAAACCCGAGAG
SUS4	AT3G43190	F	GCTATGACCTGTGGGTTACCGA
		R	CAGCTGCCTTGTCACCATGA
SUS5	AT5G37180	F	GGTTTCCACATTGACCCGAG
		R	GCGTTGTAGCCCTTCATTCG
SUS6	AT1G73370	F	AGAAGGCTTGCTTGCAGCTG
		R	TCCTTGGCTATGTTGTTGTCCA

Cloning primers:

AT1G35580g-attB1			GGGGACAAGTTTGTACAAAAAAGCAGGCTAT GTAGTATGATGCAGATCGGTACGAATC

AT1G35580g-attB2	GGGGACCACTTTGTACAAGAAAGCTGGGTC GAGTTGTGGCCAAGACGCAGATC
AT4G09510g-attB1	GGGGACAAGTTTGTACAAAAAAGCAGGCTAT TATCAACTCTAACAAGCTCAGGAAGTTG
AT4G09510-attB2	GGGGACCACTTTGTACAAGAAAGCTGGGTC GCAAGTCCATGAAGCAGATCTCTTG
AT4G34860-attB1	GGGGACAAGTTTGTACAAAAAAGCAGGCTAT ATGTCGAGTTTTAATCTGAGTGTAG
AT4G34860-attB2	GGGGACCACTTTGTACAAGAAAGCTGGGTC ACAAGTCCAGGAGTTGGATCTTCTC
AT1G22650-attB1	GGGGACAAGTTTGTACAAAAAAGCAGGCTAT ATGGAAGGAGTAAACTCTTCAAGC
AT1G22650-attB2	GGGGACCACTTTGTACAAGAAAGCTGGGTC AGTCCAAGAATAAGATCTCTTAATG
AT1G72000-attB1	GGGGACAAGTTTGTACAAAAAAGCAGGCTAT ATGTCTTTAAGCCTTTACGACAGTG
AT1G72000-attB2	GGGGACCACTTTGTACAAGAAAGCTGGGTC AGTCCAAGAAGAAGATCTTCTAAG

Genotyping primers:

SALK_095807_LP (At1g35580)	TATTGAATTTGAGTGGAGGC
SALK_095807_LP (At1g35580)	TGTAGACTGGCATAAGAACAG
SAIL_518_D02_LP (At4g09510)	ACCTTCCTCCATTTCTTTGGTTTTAATG
SAIL_518_D02_RP (At4g09510)	ACCAGACTAACAAGCTTACCAGTCC
SALK_131881_LP (At1g72000)	ACGTGGATCTTGTTTTGGTTC
SALK_131881_RP (At1g72000)	TTTTGGCATCGAACTTTTGAC
WiscDsLox466C11_LP (At1g22650)	TTTTTCATCCTCTTTTGTGGAAG
WiscDsLox466C11_RP (At1g22650)	CTTTTGGGATCTAAAGGCGTG
SALK_097137_LP (At4g34860)	GCCTCACCAACCATAGGATG
SALK_097137_RP (At4g34860)	CGTTTCGTCTCTCTCTCTCTCG

Table S2. Transcript levels for cytosolic invertases in roots[a]

Tissue	*CINV1* At1g35580	*CINV2* At4g09510	*INVB* At4g34860	*INVD* At1g22650	*INVF* At1g72000
longitudinal zone 1 [b]	544 ± 0[c]	41 ± 2	84 ± 17	10 ± 1	10 ± 7
longitudinal zone 2	753 ± 40	37 ± 2	158 ± 5	12 ± 4	5 ± 0
longitudinal zone 3	581 ± 22	43 ± 0	122 ± 16	32 ± 3	6 ± 2
longitudinal zone 4	507 ± 31	37 ± 6	86 ± 4	18 ± 2	8 ± 3
epidermis and lateral root cap	525 ± 63	46 ± 5	84 ± 3	7 ± 4	7 ± 2
columella root cap	924 ± 16	41 ± 9	113 ± 17	25 ± 2	8 ± 7
cortex	1015 ± 129	52 ± 4	221 ± 9	15 ± 1	3 ± 1
endodermis and quiescent center	627 ± 144	52 ± 9	102 ± 9	76 ± 11	2 ± 0
stele	629 ± 53	72 ± 2	163 ± 32	18 ± 14	9 ± 2
protophloem	367 ± 112	41 ± 6	97 ± 9	4 ± 2	4 ± 3
whole root	864 ± 75	28 ± 4	92 ± 1	13 ± 3	1 ± 0

[a]Data are from www.bar.utoronto.ca, extracted from Dinneny *et al.* (2008). Seedlings were grown for five days on 1x Murashige and Skoog salt mixture, 1% agar, 1% sucrose. Cell type or section-specific data were generated by fluorescence-activated cell sorting or sectioning of roots, followed by RNA extraction and microarray analysis.

[b]Longitudinal zone 1 (~150 µm) is from the root tip to the point at which shape changes from conical to cylindrical. Zone 2 (~200 µm) is from the top of zone 1 to the top of the meristematic zone. Zone 3 (~200-300 µm) is from the top of zone 2 to the region where root hairs emerge. Zone 4 (1 mm) is the region above zone 3.

[c]Values are GCOS expression signal, and are means ± SD of measurements on two or three biological replicates.

Table S3. Leaf area and stomatal density in wild-type and mutant shoots.

	Wild-type	*cinv1 cinv2*
Leaf area[a,b] (mm^2)	339 ± 41	153 ± 34
Stomata adaxial[a,c] (number mm^{-2})	86 ± 1	110 ± 1
Stomatal abaxial[a,c] (number mm^{-2})	125 ± 2	144 ± 2

[a]Leaf area and stomatal density were measured on fully-expanded leaf 6 of 35-day-old plants.
[b]For leaf area, values are from measurements on eight plants ± SD. Wild-type and mutant values are statistically significantly different (Student's T-test, P<0.0001).
[c]For stomatal density, values are means ± SD of measurements on fully-expanded leaf 6 of eight plants per genotype. For each leaf, stomata were counted on ten images, each of 0.138 mm^2 of leaf surface. Wild-type and mutant values are statistically significantly different for both adaxial and abaxial surfaces (Student's T-test, P<0.01).

Table S4. Metabolite contents of wild-type and mutant shoots.

Plants were grown for 35 days on compost in 12-h photoperiods with a light intensity of 160 µmol quanta m^{-2} s^{-1}.

Shoots end of day	Wild-type	*cinv1 cinv2*
	µmol g^{-1} FW	
Glucose	0.75 ± 0.14	2.59 ± 0.59***[b]
Fructose	0.30 ± 0.05	0.80 ± 0.13***
Sucrose	2.02 ± 0.20	3.34 ± 0.19***
Starch	68.0 ± 5.2	90 ± 5.4*
	nmol g^{-1} FW	
Trehalose 6P	0.21 ± 0.06	0.50 ± 0.06***
Glucose 6P	155 ± 21	50 ± 16***
Glucose 1P	33.2 ± 8.6	25.8 ± 2.8
Fructose 6P	52.4 ± 9.4	18.5 ± 7.0**
3-PGA	196 ± 58	116 ± 28*
PEP	18.5 ± 6.2	7.1 ± 2.7*
Pyruvate	113 ± 38	78 ± 9
Shoots end of night		
	µmol g^{-1} FW	
Glucose	0.17 ± 0.11	0.27 ± 0.18
Fructose	0.09 ± 0.12	0.10 ± 0.03
Sucrose	0.88 ± 0.20	2.34 ± 0.41**
Starch	4.3 ± 0.4	39.6 ± 7.5*

	nmol g^{-1} FW	
Trehalose 6P	0.031 ± 0.004	0.161 ± 0.022***
Glucose 6P	78 ± 9	65 ± 17
Glucose 1P	28.6 ± 5.5	26.8 ± 6.2
Fructose 6P	18.1 ± 2.4	16.6 ± 3.8
3-PGA	38 ± 9	53 ± 24
PEP	7.4 ± 1.9	8.5 ± 8.7
Pyruvate	33 ± 2	38 ± 8

Figure S1

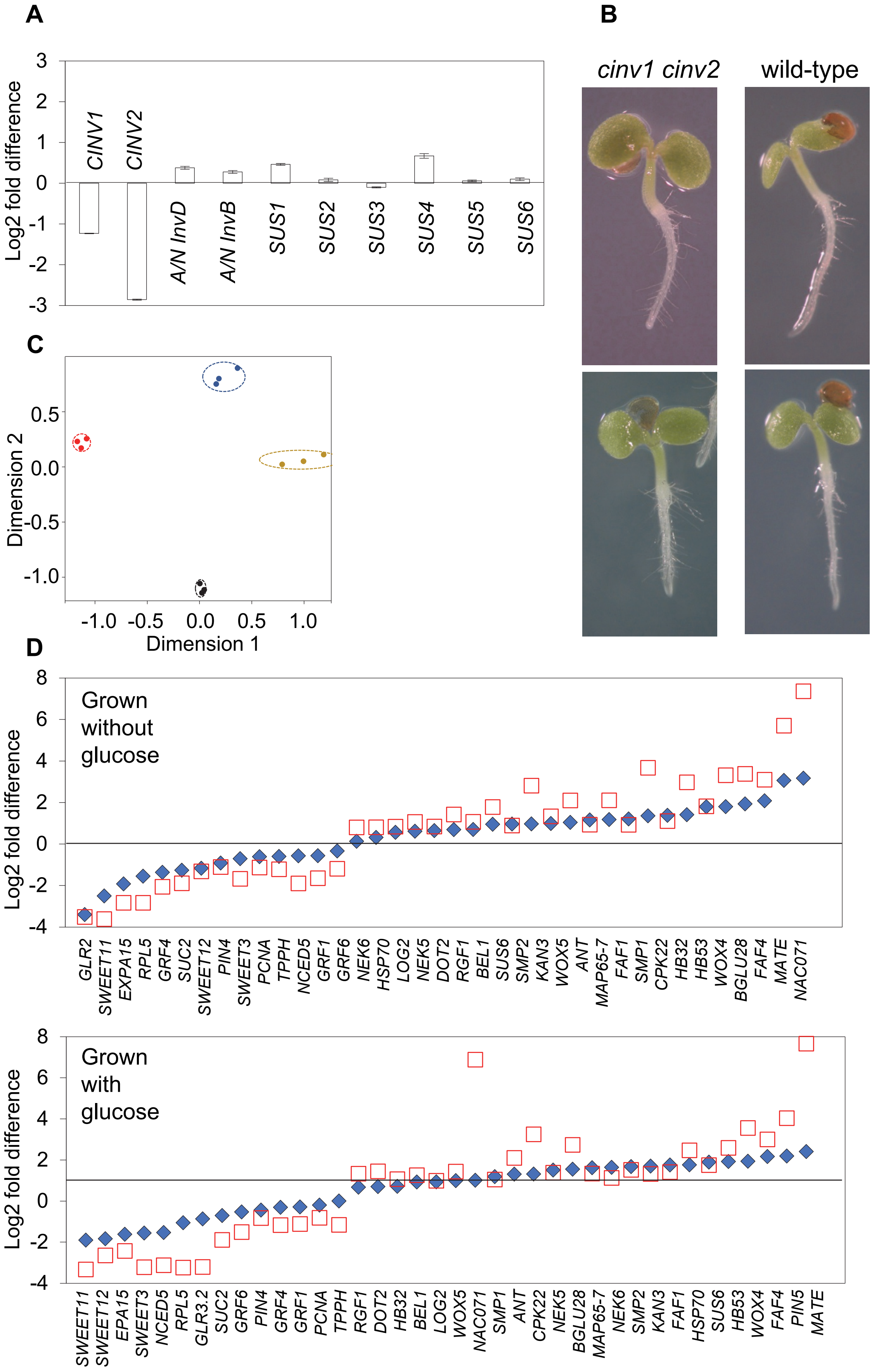
A
B
C
D
Log2 fold difference
CINV1
CINV2
A/N InvD
A/N InvB
SUS1
SUS2
SUS3
SUS4
SUS5
SUS6
cinv1 cinv2
wild-type
Dimension 2
Dimension 1
Grown without glucose
GLR2
SWEET11
EXPA15
RPL5
GRF4
SUC2
SWEET12
PIN4
SWEET3
PCNA
TPPH
NCED5
GRF1
GRF6
NEK6
HSP70
LOG2
NEK5
DOT2
RGF1
BEL1
SUS6
SMP2
KAN3
WOX5
ANT
MAP65-7
FAF1
SMP1
CPK22
HB32
HB53
WOX4
BGLU28
FAF4
MATE
NAC071
Grown with glucose
SWEET11
SWEET12
EPA15
SWEET3
NCED5
RPL5
GLR3.2
SUC2
GRF6
PIN4
GRF4
GRF1
PCNA
TPPH
RGF1
DOT2
HB32
BEL1
LOG2
WOX5
NAC071
SMP1
ANT
CPK22
NEK5
BGLU28
MAP65-7
NEK6
SMP2
KAN3
FAF1
HSP70
SUS6
HB53
WOX4
FAF4
PIN5
MATE

Fig. S1. Transcript levels for sucrose metabolizing enzymes in *cinv1 cinv2* roots, and validation of RNA-seq data. (A) Levels of transcripts of cytosolic INV and SUS in 14-day-old seedlings, measured by q-PCR. Values are expressed as log2 fold difference in transcript abundance in *cinv1 cinv2* relative to wild-type. Values are means of measurements on three independent biological replicates ± SD. There is essentially no difference between 14-day-old mutant and wild-type roots with respect to transcript levels for *SUS1*, *SUS2*, *SUS5* and *SUS6*, and only a modest elevation (≤1.6-fold) of transcripts for *SUS1*, *SUS4*, *A/N-InvB and A/N-InvD.* For four-day-old plants RNA-seq data (Supplementary Table S5) show that transcript levels for *SUS2*, *SUS4*, *A/N-InvB* and *A/N-InvD*, a vacuolar INV and two mitochondrial neutral INV were slightly reduced and transcript levels of *cwINV1* and of the phloem-specific protein *SUS6* were higher in mutant than in wild-type roots; (B) Photographs of four-day-old mutant and wild-type seedlings grown without glucose. All images are at the same magnification; (C) Multi-dimensional scaling plot visualizing the distance between the libraries in the RNA-seq experiment, based on the 500 tags with the largest variation between the four treatments. The distance between each pair of libraries is equivalent to the square root of the common dispersion between these two libraries. Libraries were from roots of four-day-old seedlings. Green symbols, wild-type grown without glucose; blue symbols, wild-type grown with 55 mM glucose; black symbols, *cinv1 cinv2* grown without glucose; red symbols, *cinv1 cinv2* grown with 55 mM glucose. For each genotype/treatment, the three points represent the three independent biological replicates (see Materials and Methods); (D) Validation of RNA-seq analysis. For 38 selected transcripts representing a wide range of different cellular functions (loci given in Supplementary Table S1 below), the graphs show log2 fold differences in transcript abundance in *cinv1 cinv2* relative to wild-type from qPCR measurements (blue diamonds) and RNA-seq (red squares: see Supplementary Table S5). For qPCR, values are means of measurements on three independent biological replicates. In all cases SD was less than 10% of the mean.

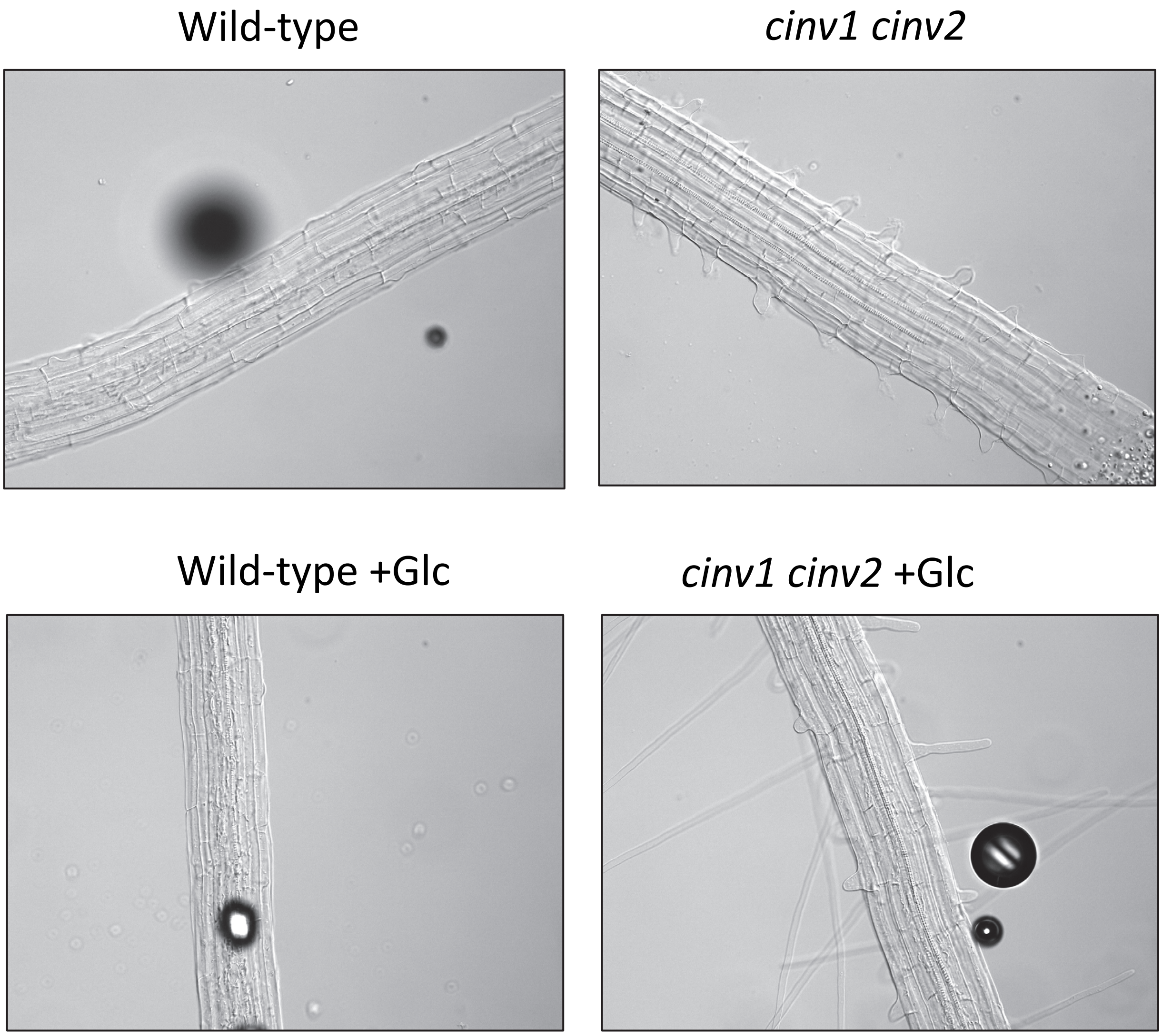

Fig. S2. Original, uncropped micrographs from which the composite Fig. 1D was assembled.

Figure S3

A

Cytosol	Chloroplast	Mitochondria
At1g35580 (*CINV1*, *A/N-InvG*)	At5g22510 (*A/N-InvE*)	At1g56560 (*A/N-InvA*)
At4g09510 (*CINV2*, *A/N-InvI*)		At3g05820 (*A/N-InvH*)
At4g34860 (*A/N-InvB*)		At3g06500 (*A/N-InvC*)
At1g22650 (*A/N-InvD*)		
At1g72000 (*A/N-InvF*)		

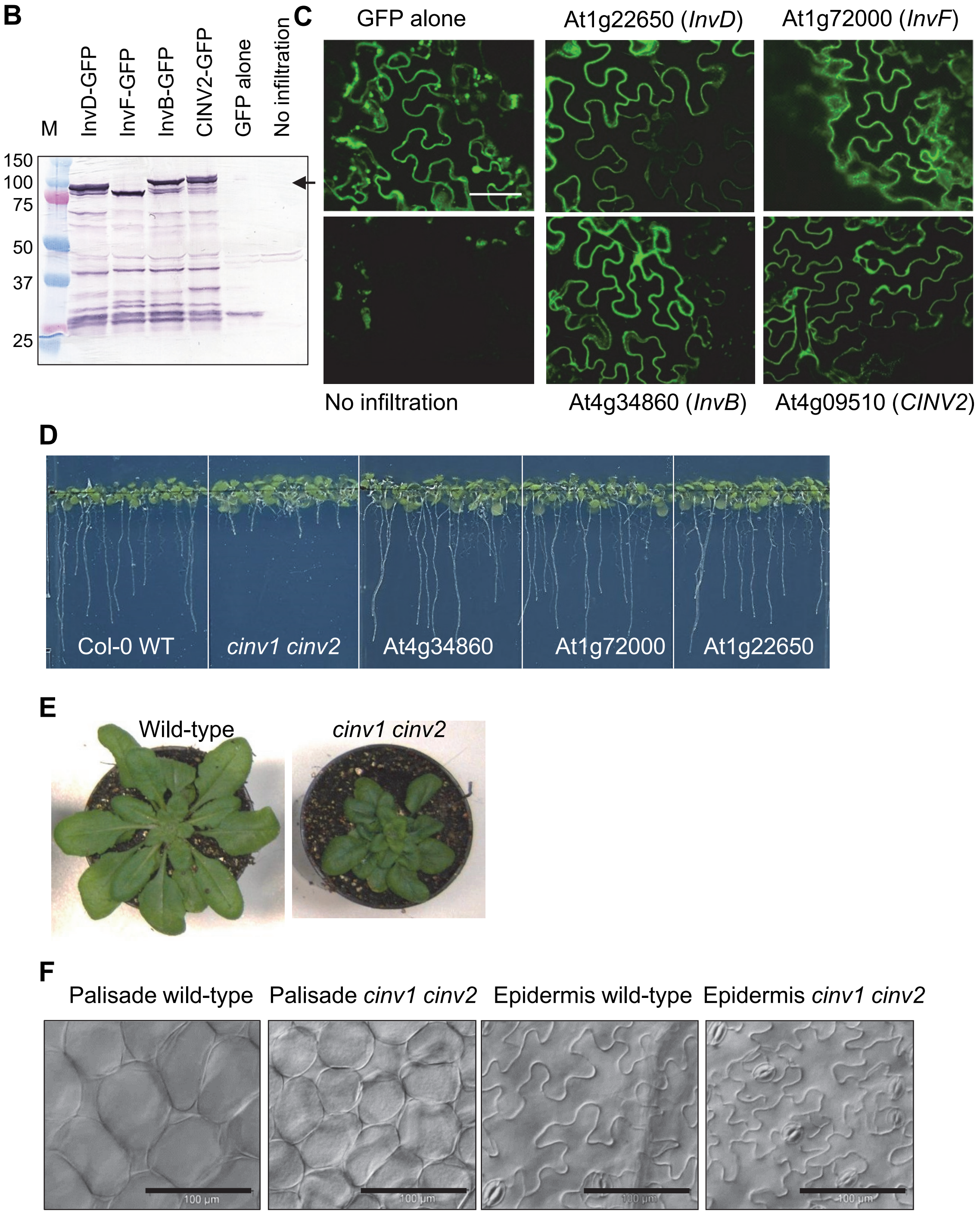

Fig. S3. Characterization of Arabidopsis neutral INV. (A) Cellular location of the neutral INVs, deduced from (B) and (for At1g35580, At5g25510, At1g56560, At3g05820 and At3g06500) from Vargas *et al*. (2008); Xiang *et al*. (2011); Martin *et al*. (2013); Battaglia *et al*. (2017); (B) Expression in *Nicotiana benthamiana* of INV-GFP translational fusions for isoforms without confirmed locations. Translational GFP fusions for 4 isoforms were transiently expressed. After 3 days, GFP fusion proteins were detected by immunoblotting SDS-PAGE gels of extracts of infiltrated regions with a commercial antiserum raised against GFP. Each lane contains extract from a single leaf, all lanes represent the same fresh weight of leaf. Lane M is molecular markers (kDa). Arrow indicates fusion proteins. Predicted masses (kDa) are: InvD-GFP 91; InvF-GFP 87; InvB-GFP 95; CINV2-GFP 94. Right lanes are transient expression of GFP alone, and an uninfiltrated region. (C) Confocal microscopy of intact leaves expressing the Inv-GFP fusion proteins shown in (B). Note that fluorescence is only in the cytosol. Control panels (left), a leaf expressing 35S::GFP (fluorescence in the cytosol and nucleus), and an uninfiltrated leaf. Images are all the same magnification, bar is 50 µm. (D) Seedling growth on vertical plates of wild-type and *cinv1 cinv2* mutants, and T-DNA insertion mutants for InvB, InvD and InvF; (E) Wild-type and *cinv1 cinv2* plants grown in soil for 35 days, 12-h photoperiods, light intensity 160 µmol quanta m^{-2} s^{-1}; (F) Abaxial epidermis and palisade mesophyll cross-sections of mature leaf 6. Bars are 100 µm.

Battaglia ME, Martin MV, Lechner L, Martínez-Noël GMA, Salerno GL. 2017. The riddle of mitochondrial alkaline/neutral invertases: A novel Arabidopsis isoform mainly present in reproductive tissues and involved in root ROS production. PLoS ONE 12:e0185286

Martín ML, Lechner L, Zabaleta EJ, Salerno GL. 2013. A mitochondrial alkaline/neutral invertase isoform (A/N-InvC) functions in developmental energy-demanding processes in Arabidopsis. Planta 237, 813-822.

Vargas WA, Pontis HG, Salerno GL. 2008. New insights on sucrose metabolism: evidence for an active A/N-Inv in chloroplasts uncovers a novel component of the intracellular carbon trafficking. Planta 227, 795-807.

Xiang L, Le Roy K, Bolouri-Moghaddam MR, Vanhaeke M, Lammens W, Rolland F, Van den Ende W. 2011. Exploring the neutral invertase–oxidative stress defence connection in *Arabidopsis thaliana.* Journal of Experimental Botany 62, 3849-3862.

Figure S4

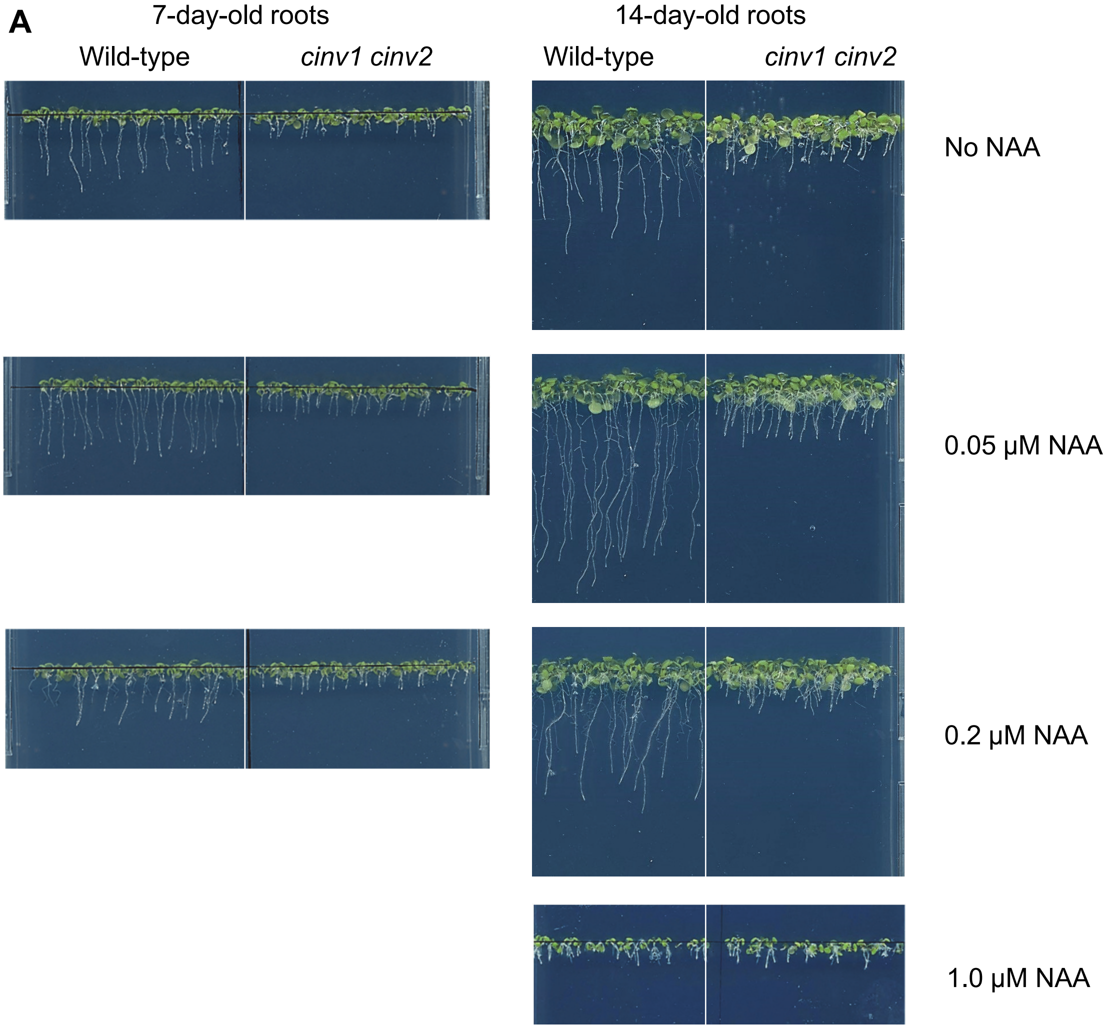
A
7-day-old roots
14-day-old roots
Wild-type
cinv1 cinv2
Wild-type
cinv1 cinv2
No NAA
0.05 μM NAA
0.2 μM NAA
1.0 μM NAA

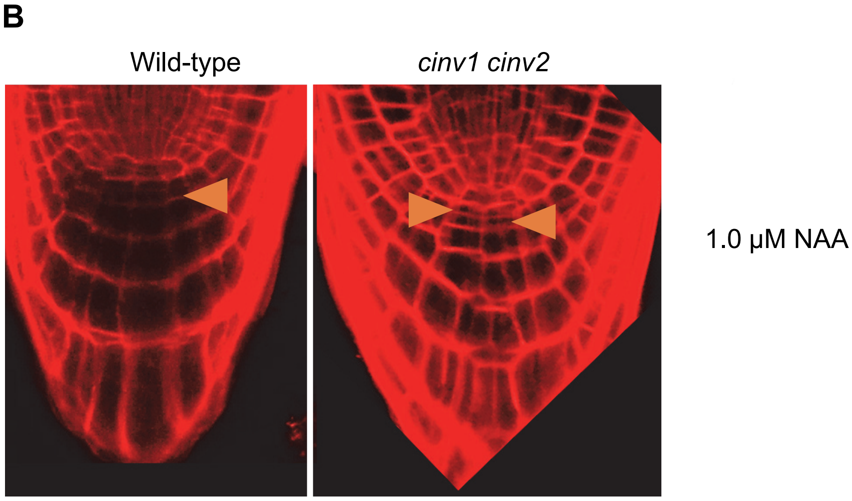
B
Wild-type
cinv1 cinv2
1.0 μM NAA

Fig. S4. Effects of auxin on *cinv1 cinv2* root elongation and developmental abnormalities. (A) Appearance of wild-type and *cinv1 cinv2* seedlings grown on vertical agar plates for 7 or 14 days in the presence of different concentrations of 1-naphthaleneacetic acid (NAA); (B) **D**ifferentiation status of columella cells in 4-day-old wild-type and *cinv1 cinv2* seedlings grown on 1 μM NAA. Roots were stained with propidium iodode. Arrows indicate columella stem cells.

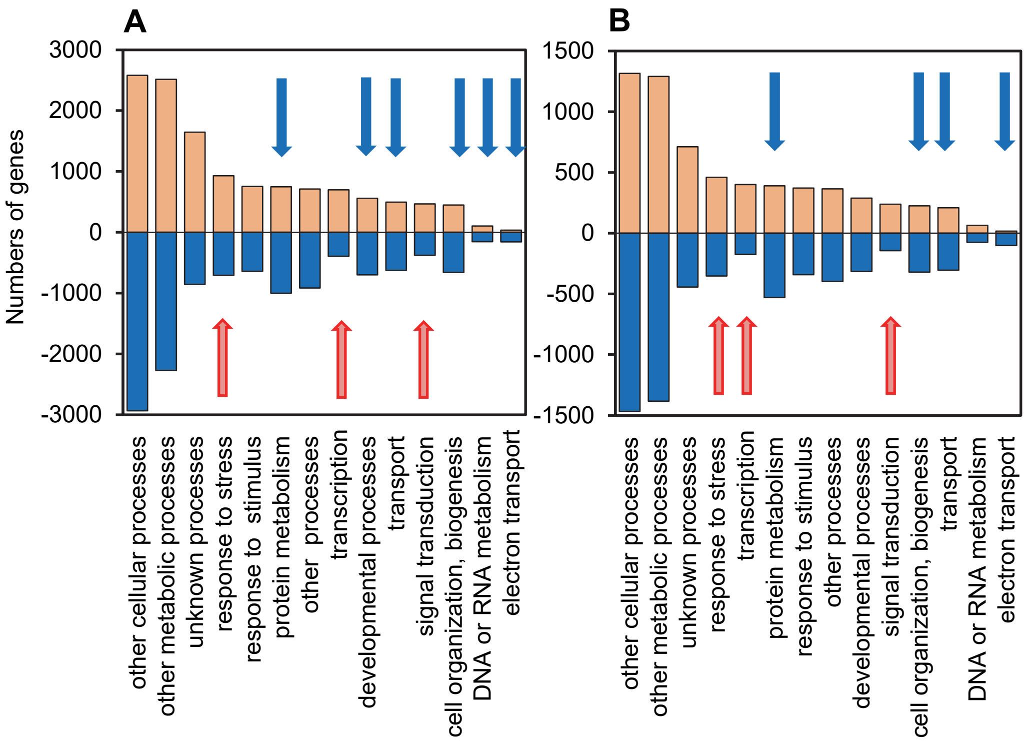

Fig. S5. Functional characterisation of genes differentially expressed in mutant and wild-type roots (DEGs) into GO-slim Biological Processes. (A) Roots of four-day old seedlings grown without 55 mM glucose; (B) Roots of four-day old seedlings grown with 55 mM glucose. Orange bars are numbers of genes in each category with higher transcript levels in mutant than in wild-type roots. Blue bars are numbers of genes in each category with lower transcript levels in mutant than in wild-type roots. Red arrows indicate some categories in which more genes are up-regulated than down-regulated in the mutant; blue arrows indicate some categories in which more genes are down-regulated than up-regulated in the mutant.

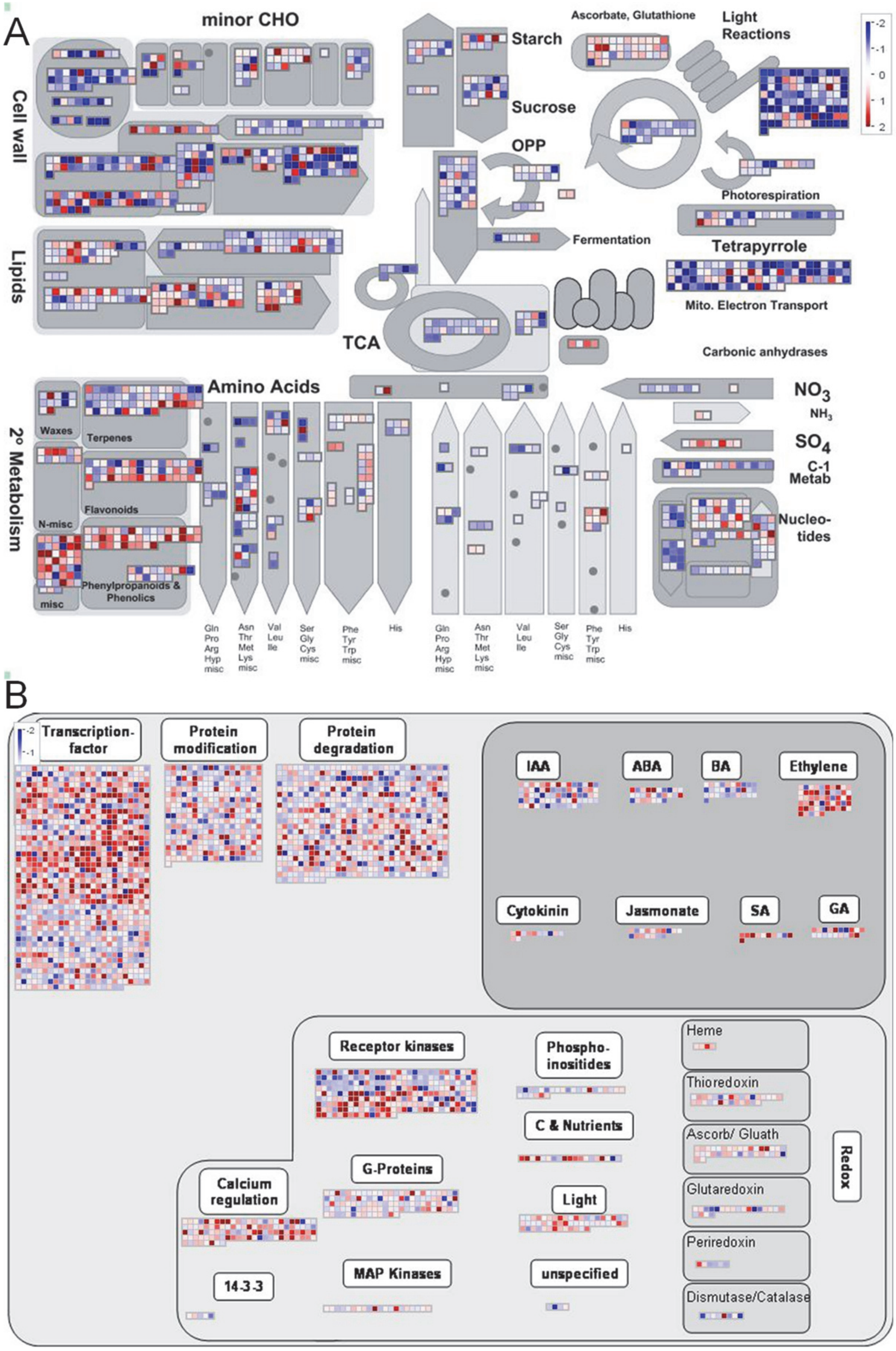

Fig. S6. MAPMAN visualization of genes differentially expressed in mutant and wild-type roots whether or not glucose was supplied. (A) Metabolism-related genes; (B) Genes involved in regulation. Blue and red boxes represent genes with reduced and elevated expression respectively, in *cinv1 cinv2* relative to wild-type roots.
